# Supplementary material for: Protein Arginine Methylation Patterns in Plasma Small Extracellular Vesicles Are Altered in Patients with Early-Stage Pancreatic Ductal Adenocarcinoma
Source: Cancers (Basel). 2024 Feb 3;16(3):654. doi: 10.3390/cancers16030654 (PMC10854811; doi:10.3390/cancers16030654)
Supplement: Supplementary file 1 [file cancers-16-00654-s001.zip › cancers-2827280-Supplemental Materials_Uncropped blots.pdf]

Figure 1A

SDMA cellular lysates

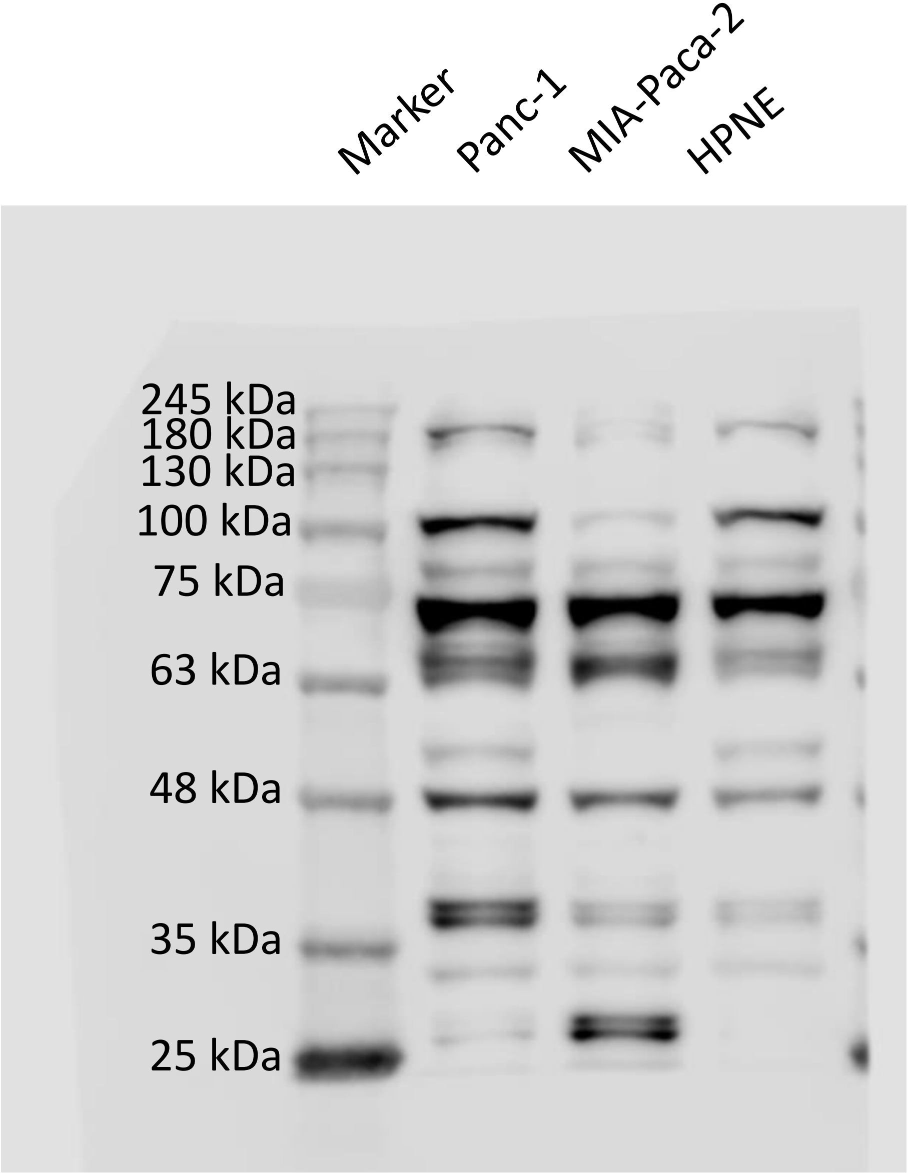

Figure 1A
GAPDH cellular
lysates

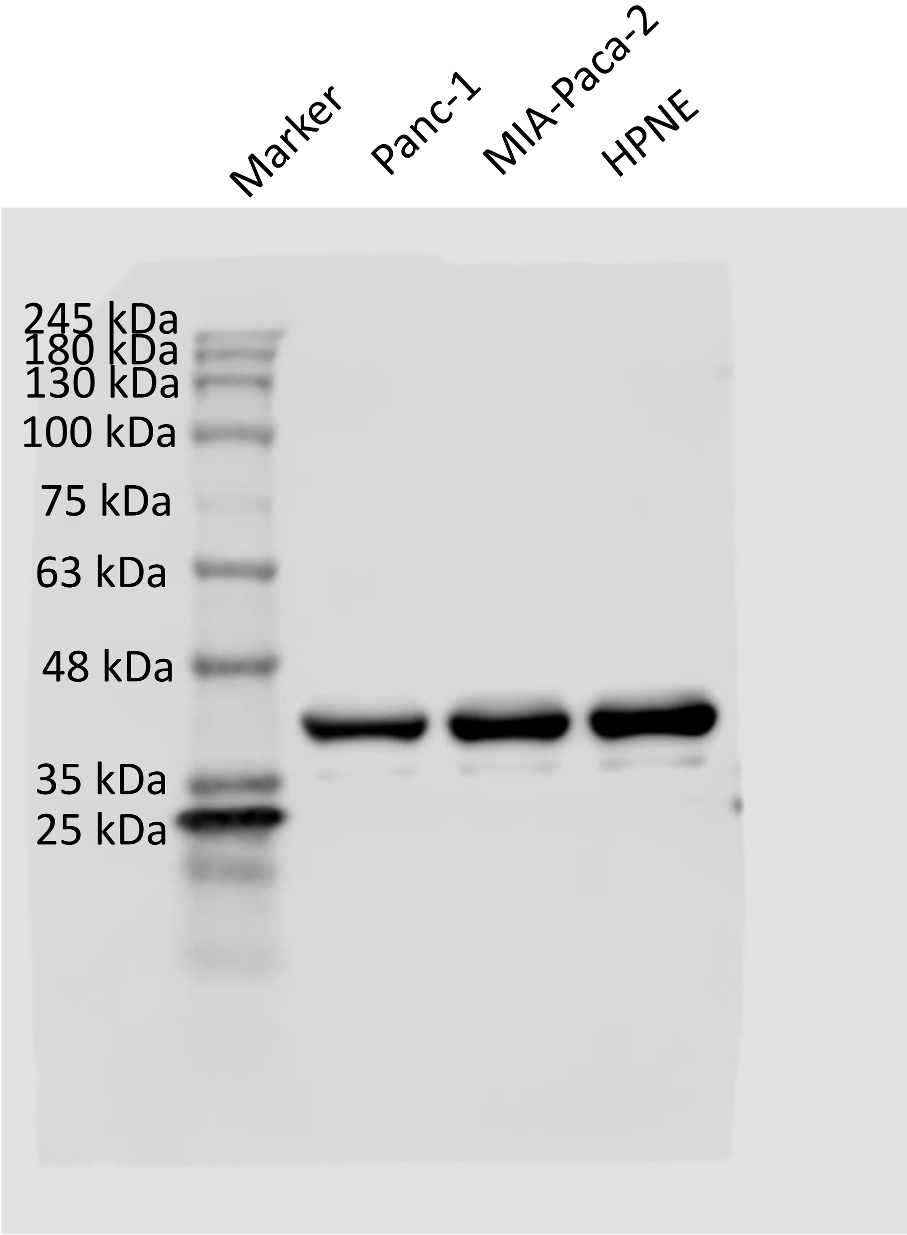

Figure 1B

MMA cellular lysates

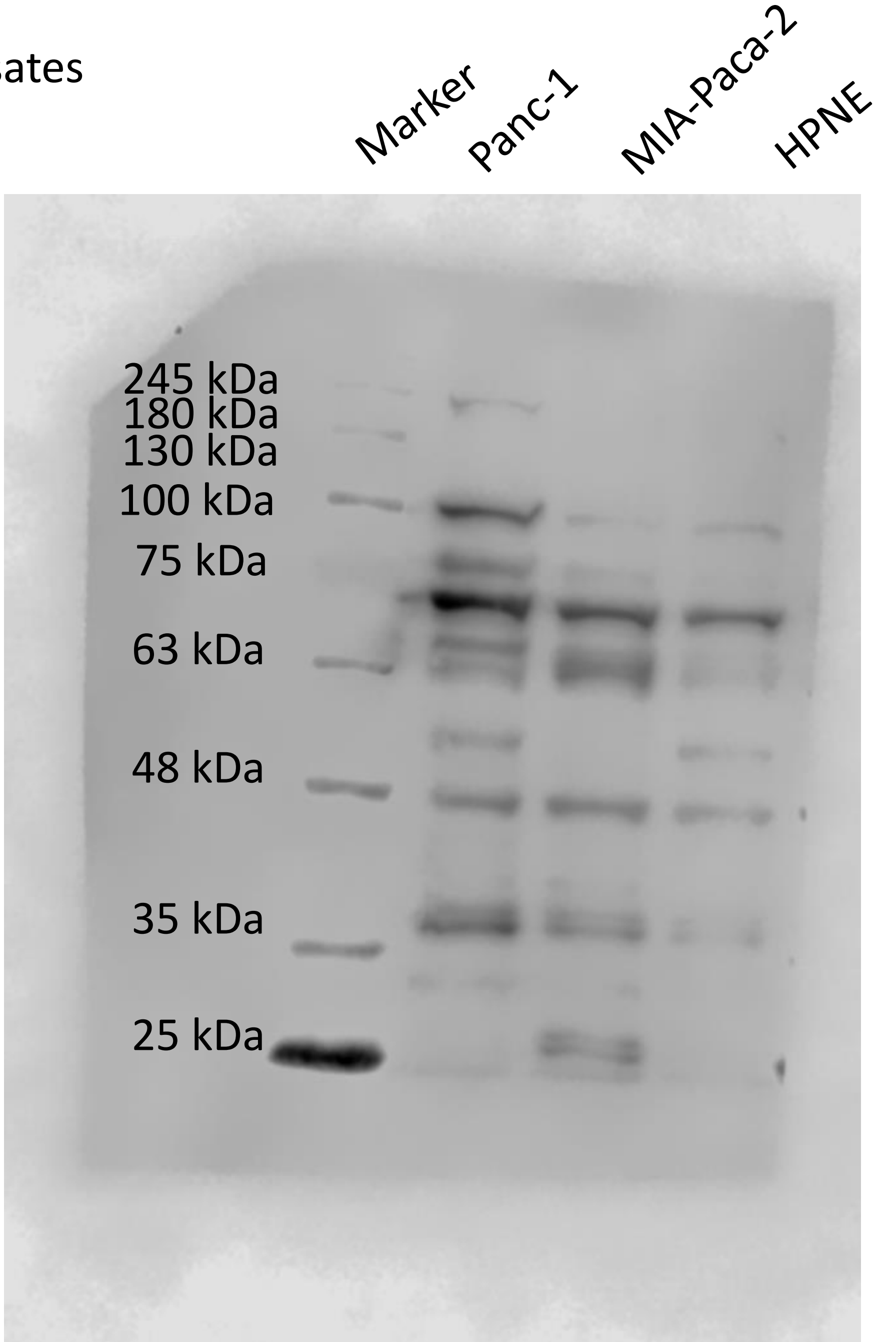

Figure 1B

GAPDH cellular lysates

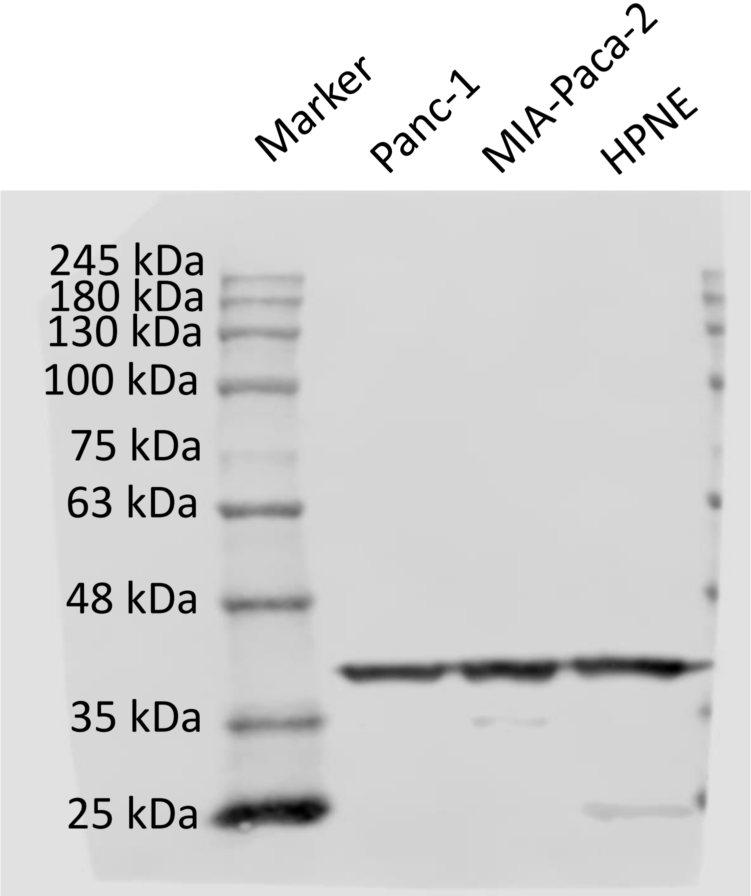

Figure 1C
SDMA
exosome
lysates

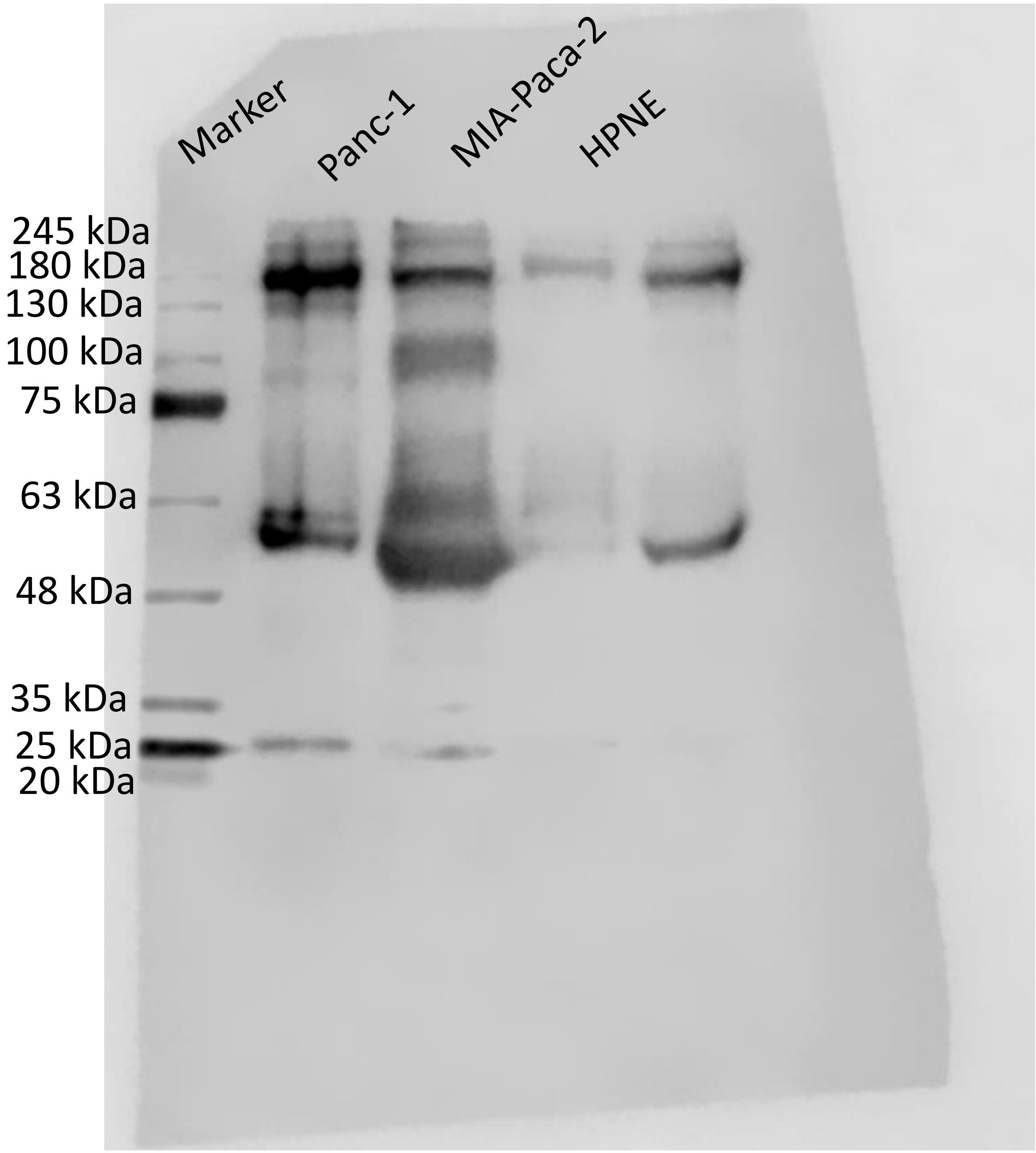

Figure 1C

GAPDH sEV lysates

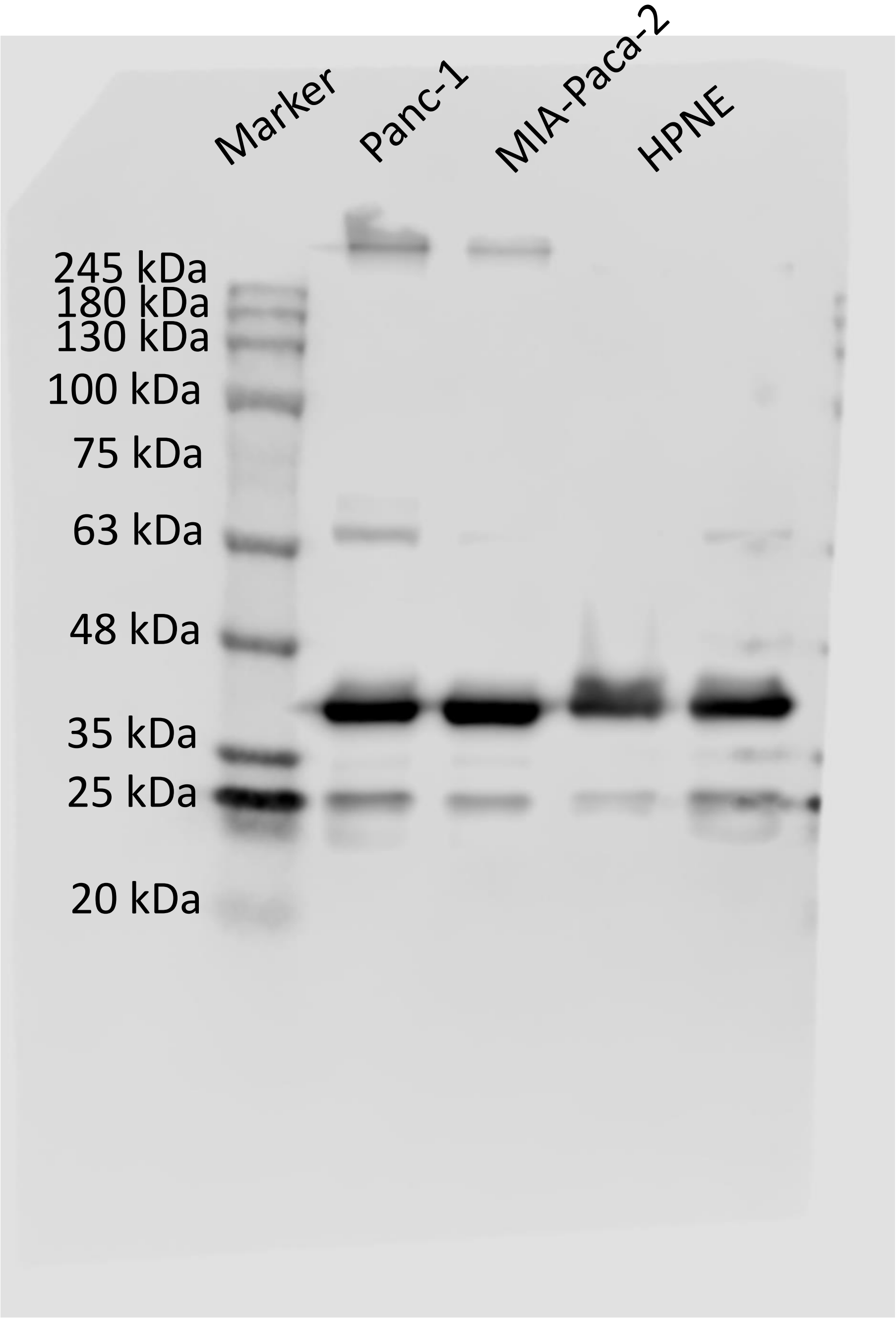

Figure 1D

MMA sEV lysates

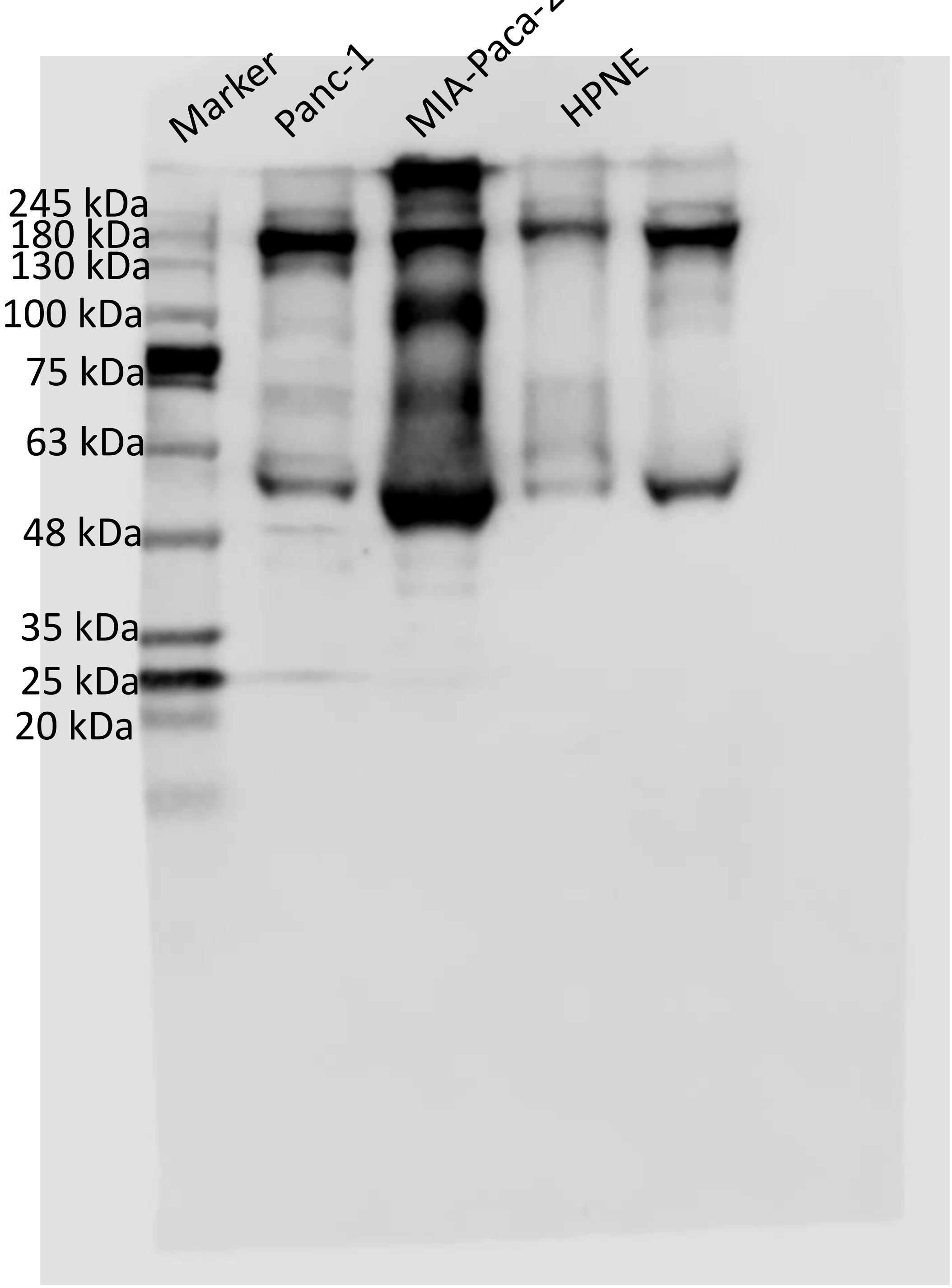

Figure 1D

GAPDH sEV lysates

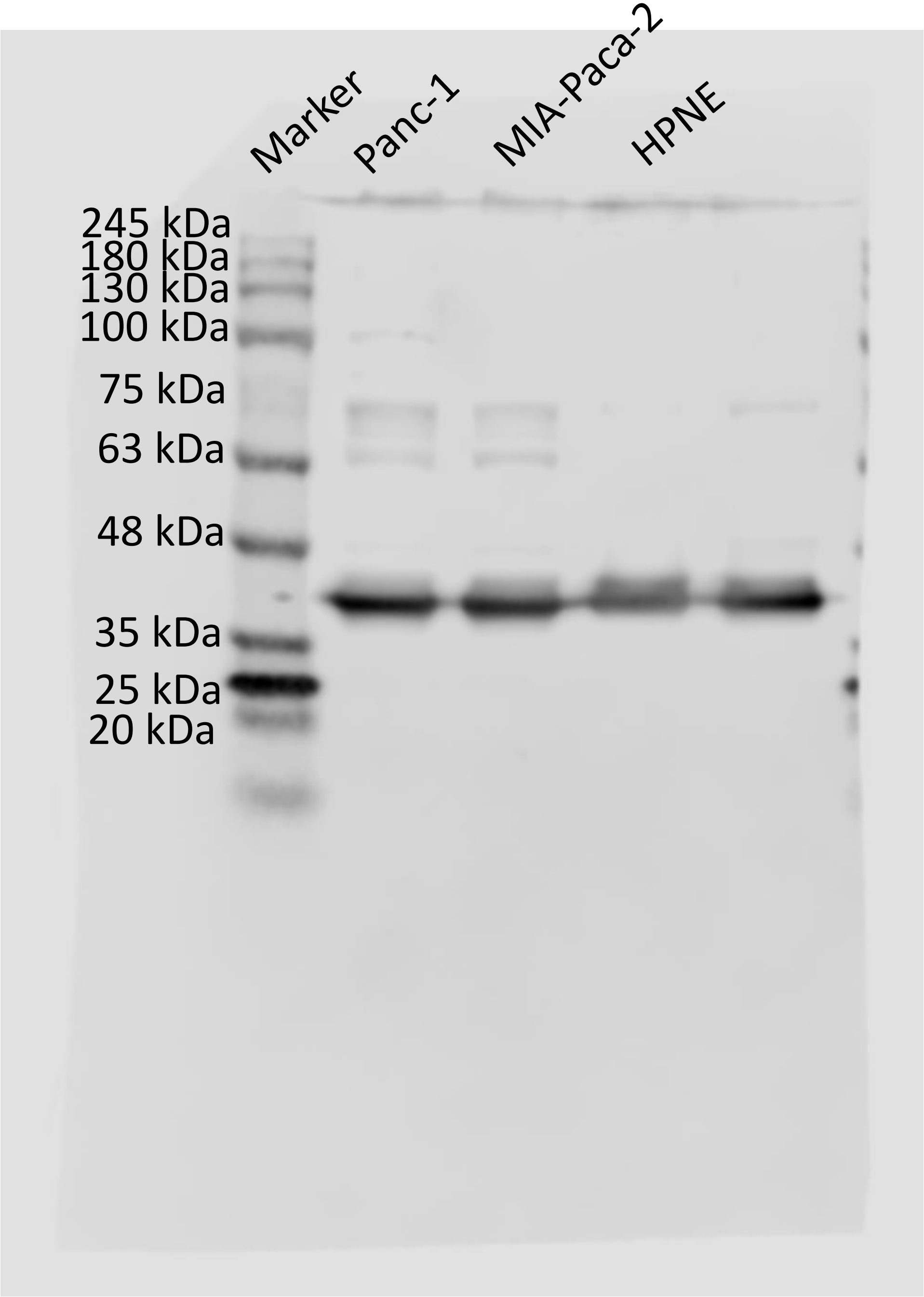

Figure 1E

Flotillin sEV lysates

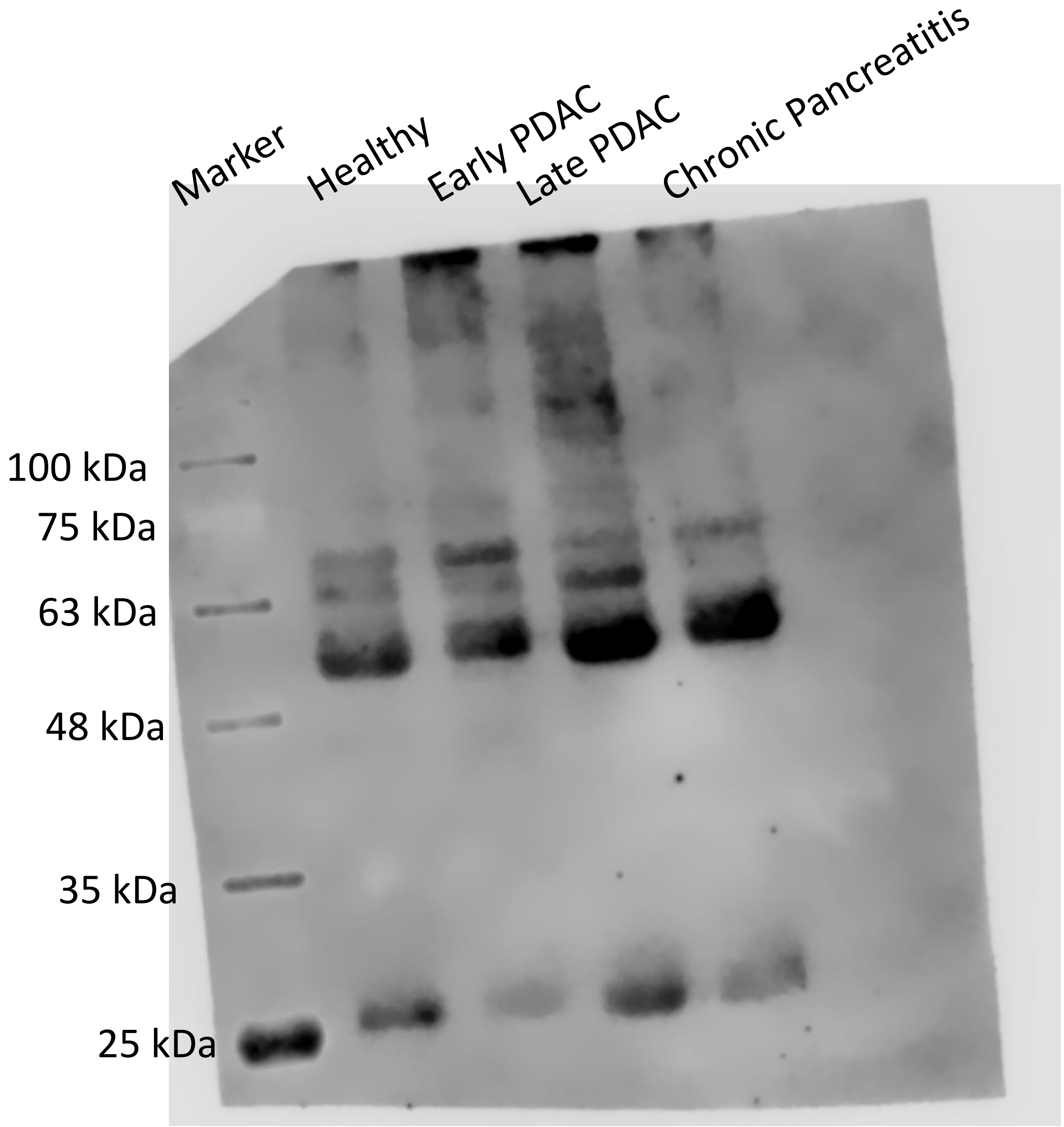

Figure 1E

CD63 sEV lysates

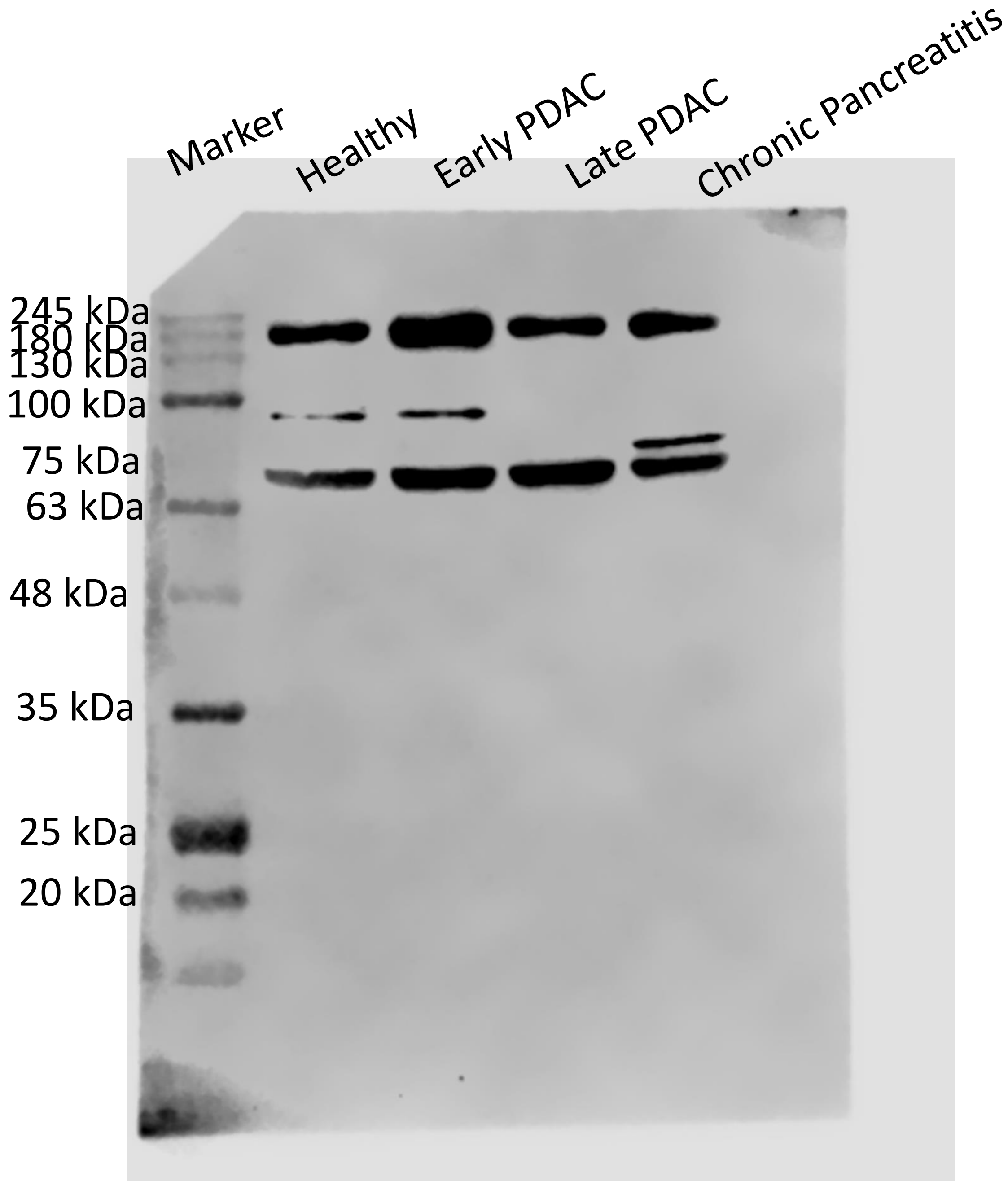

Figure 1E

CD9 sEV lysates

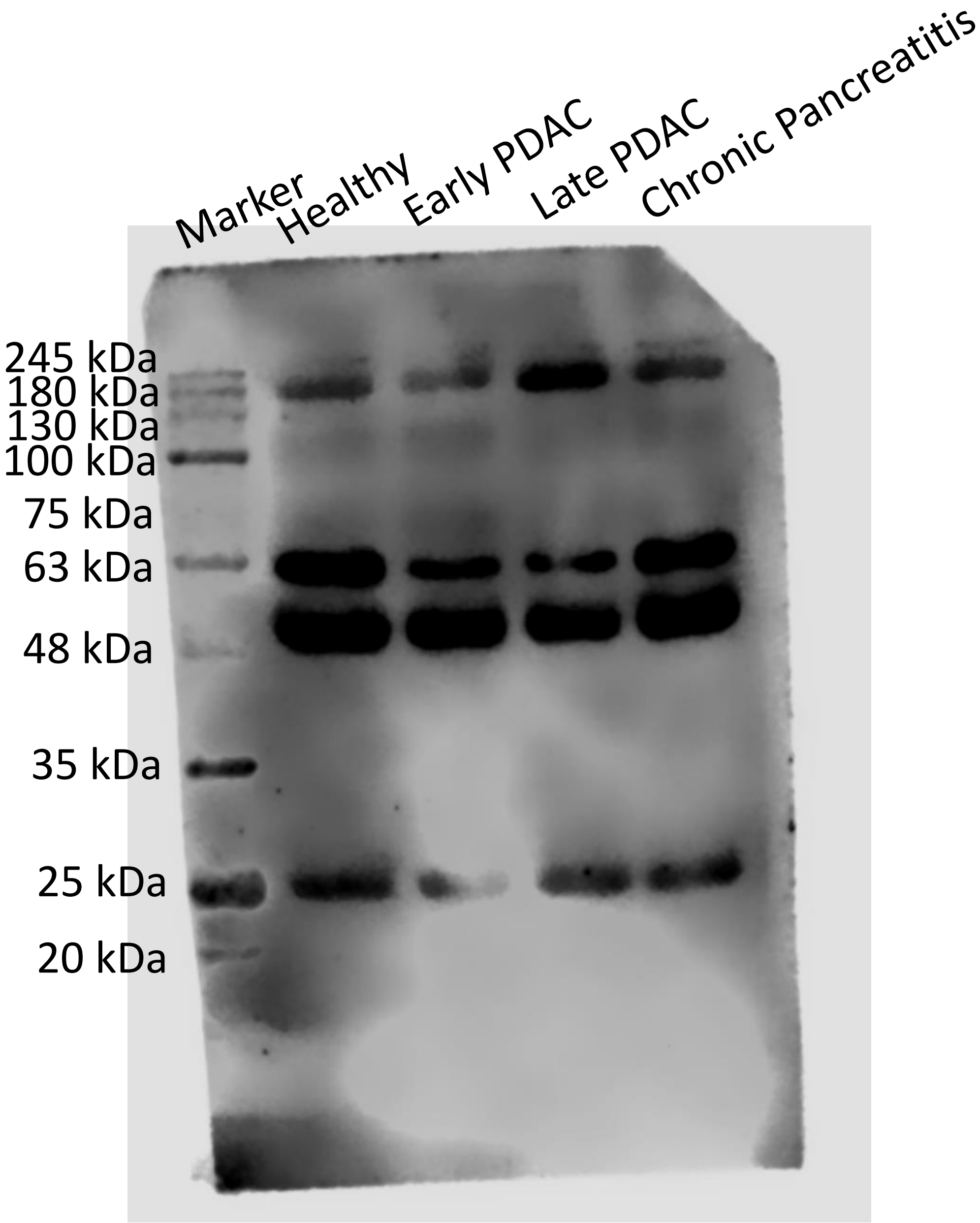

Figure 1E
Calnexin sEV lysates

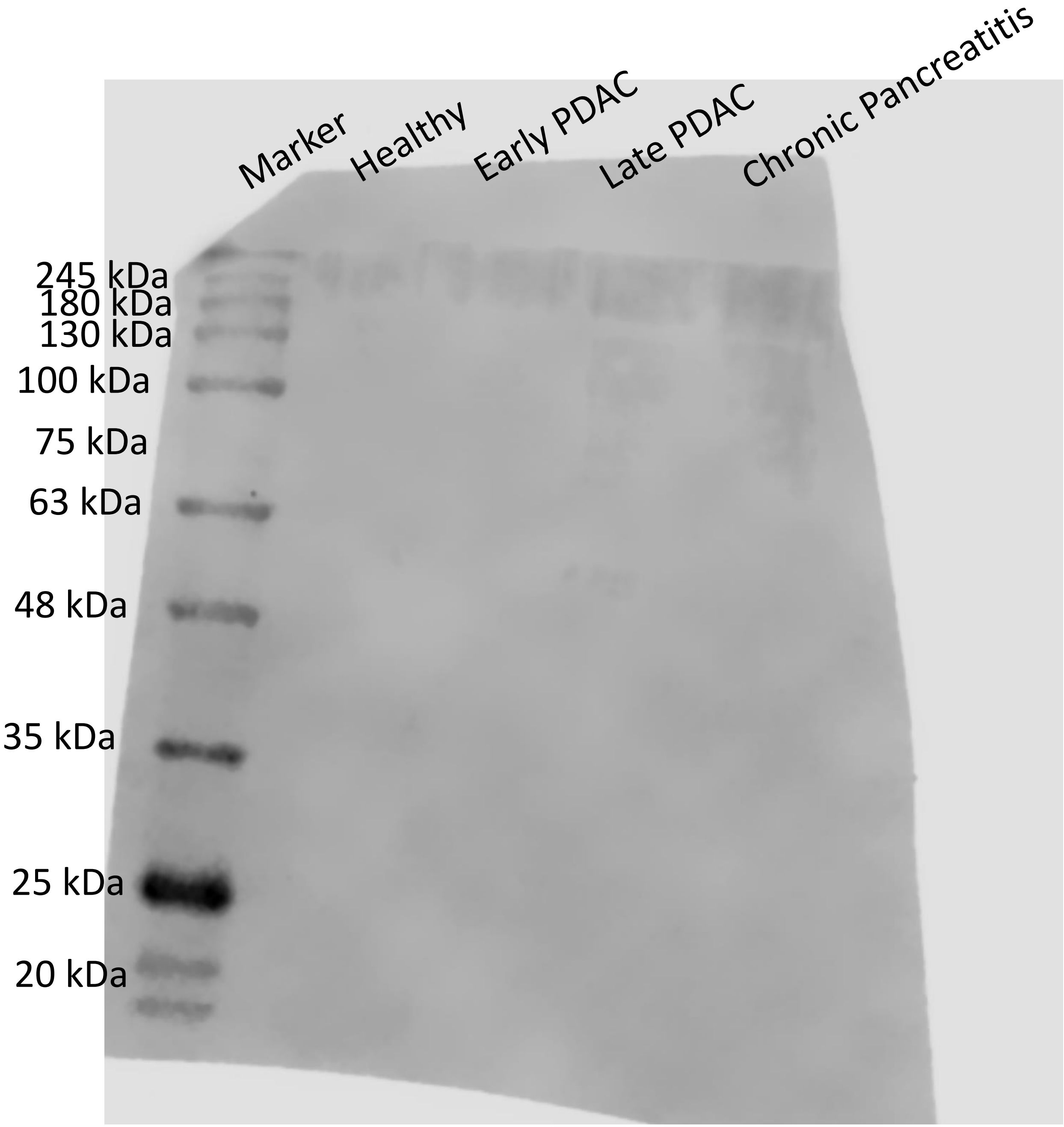

Figure 2A and 2B Coomassie

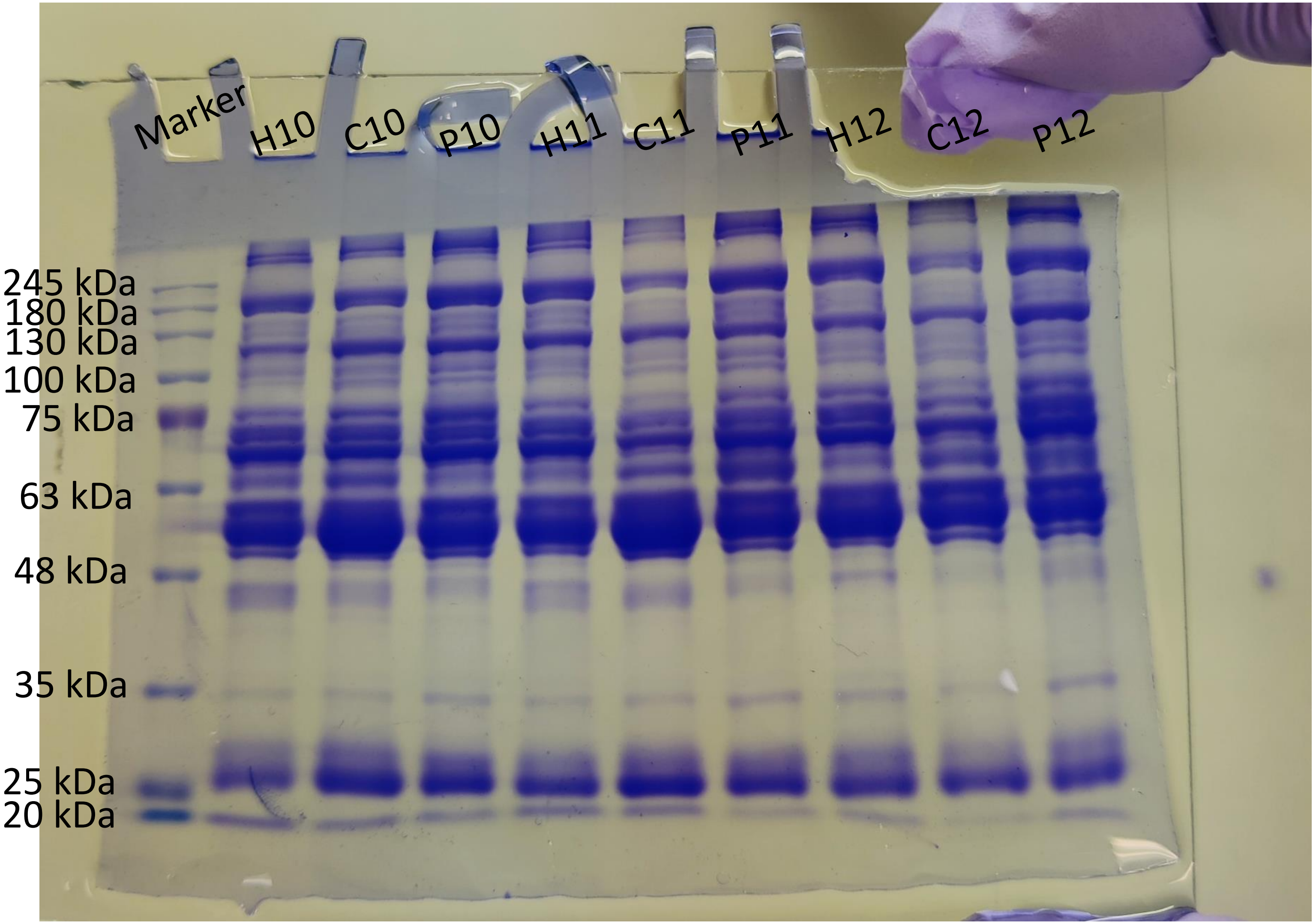

Figure 2A MMA

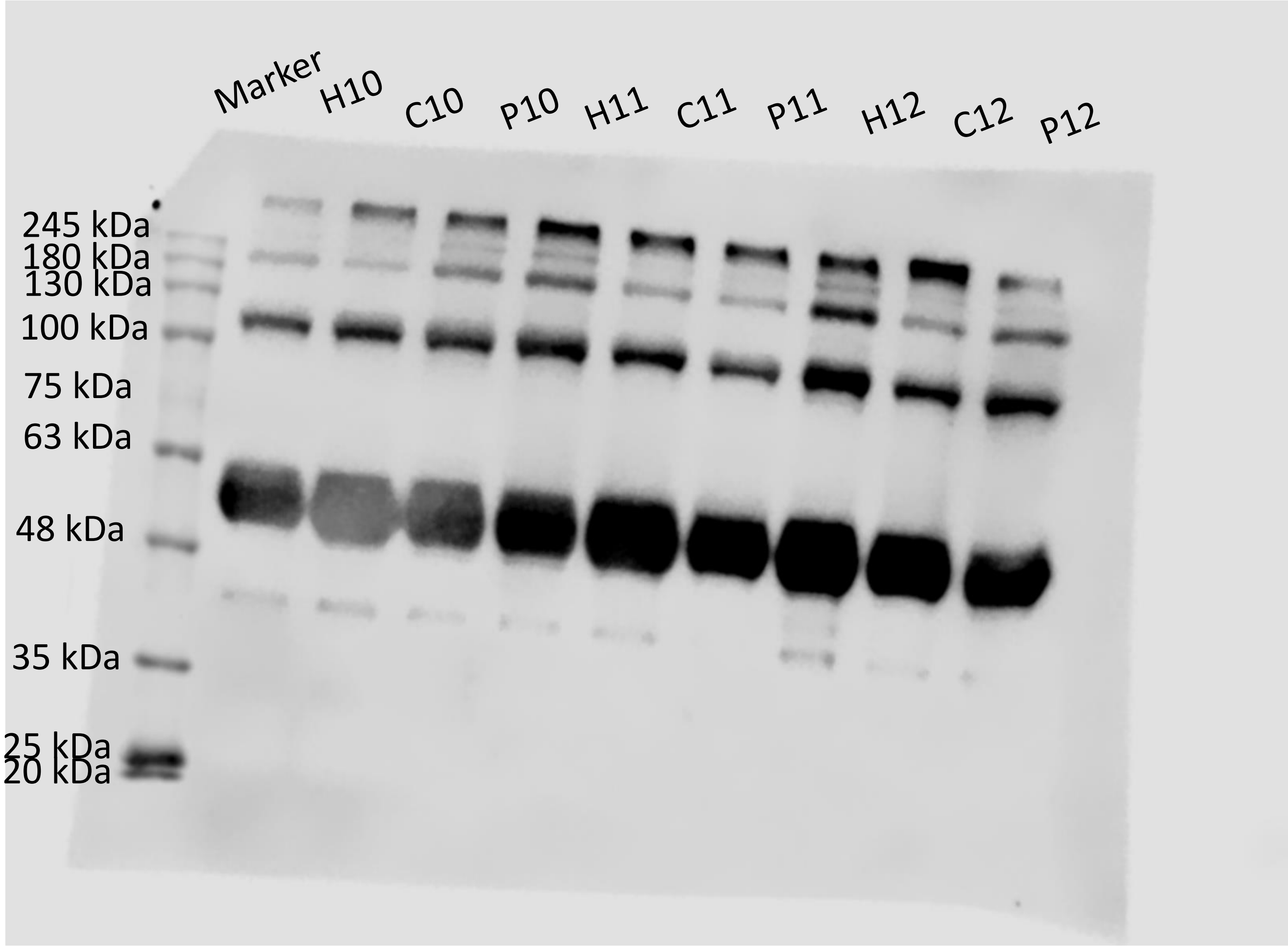

Figure 2B SDMA

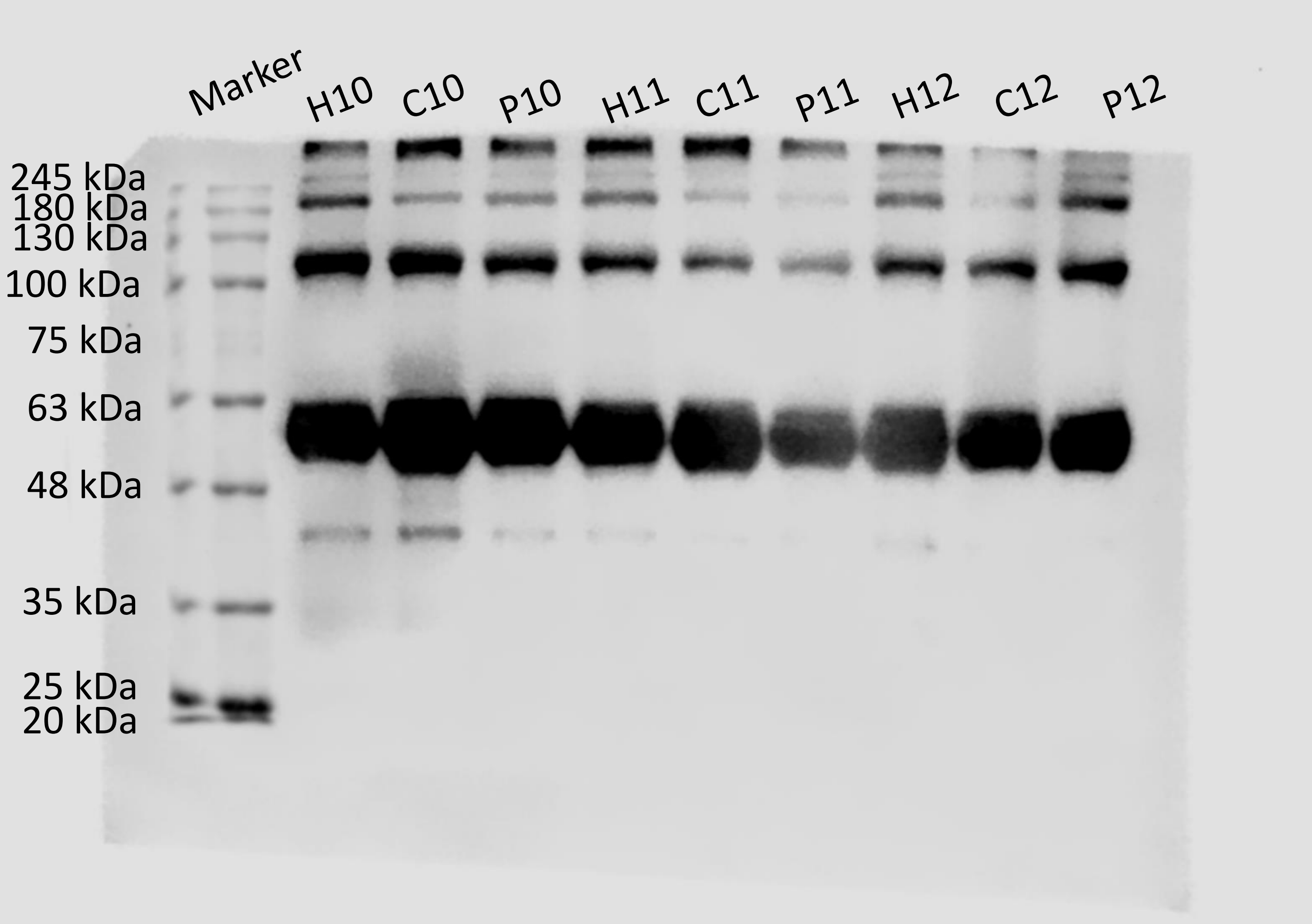

Figure 2C

Coomassie

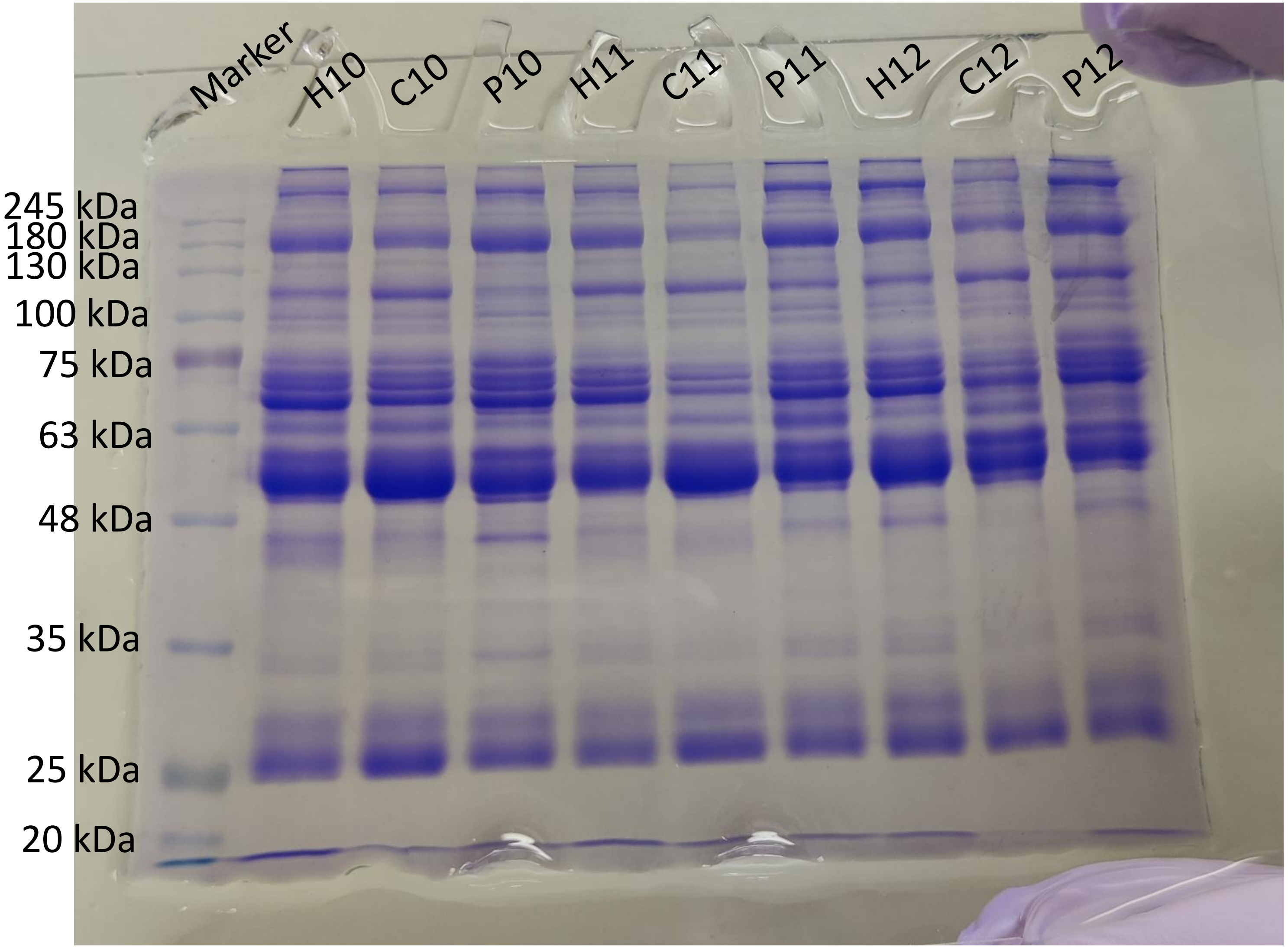

Figure 2C

ADMA

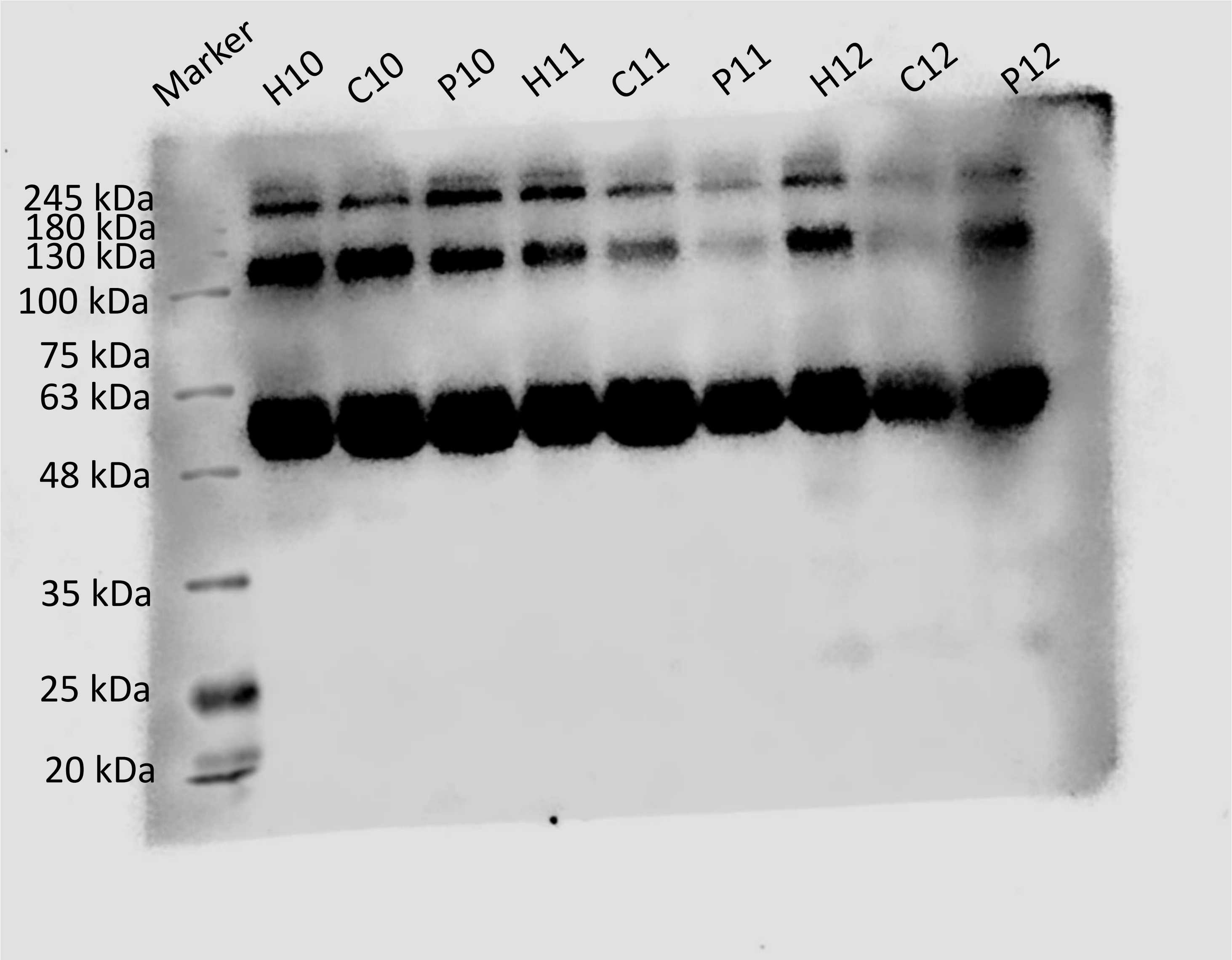

Figure 3A LPDAC
Coomassie

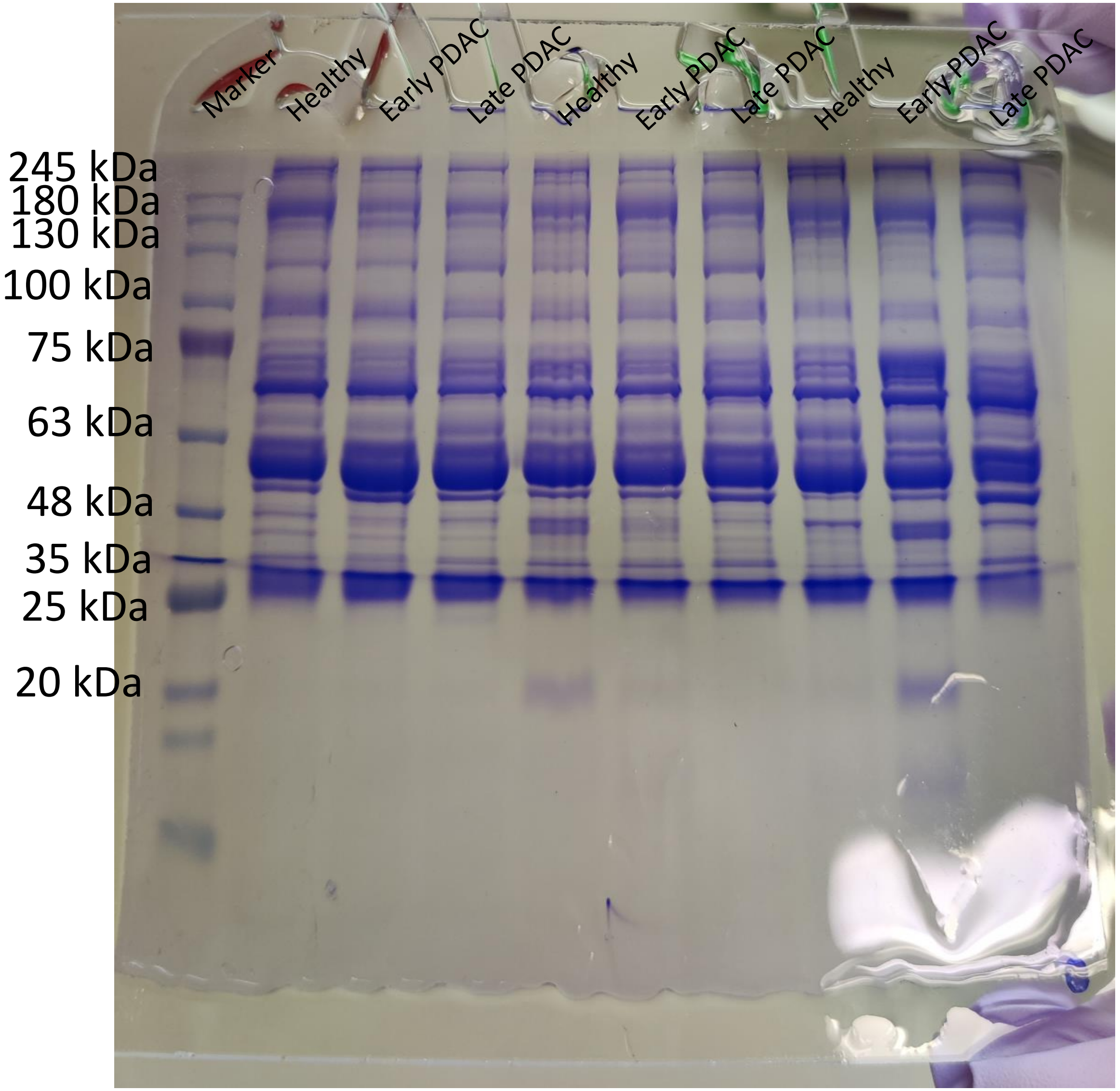

Figure 3A MMA LPDAC

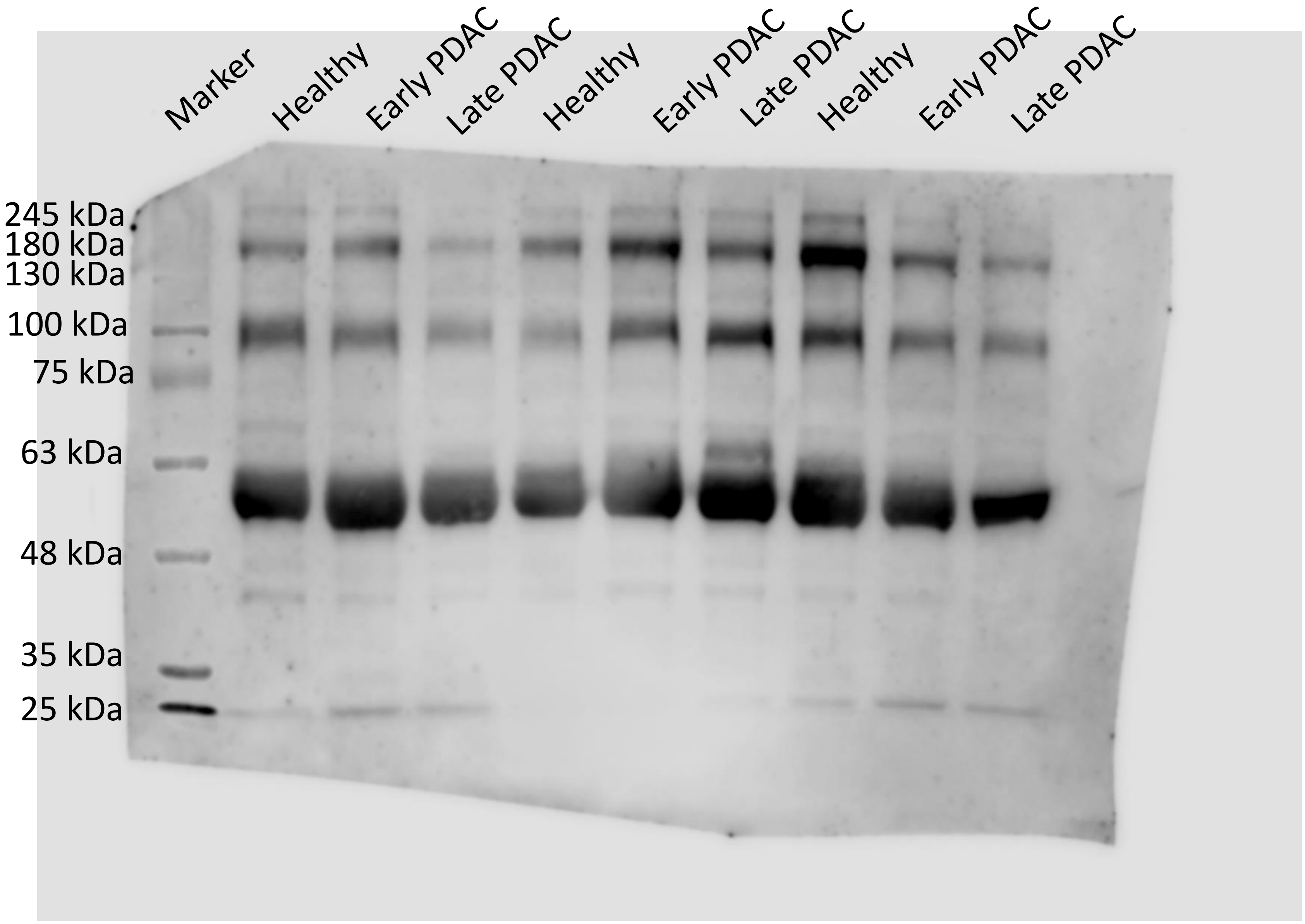

Figure 3A SDMA LPDAC

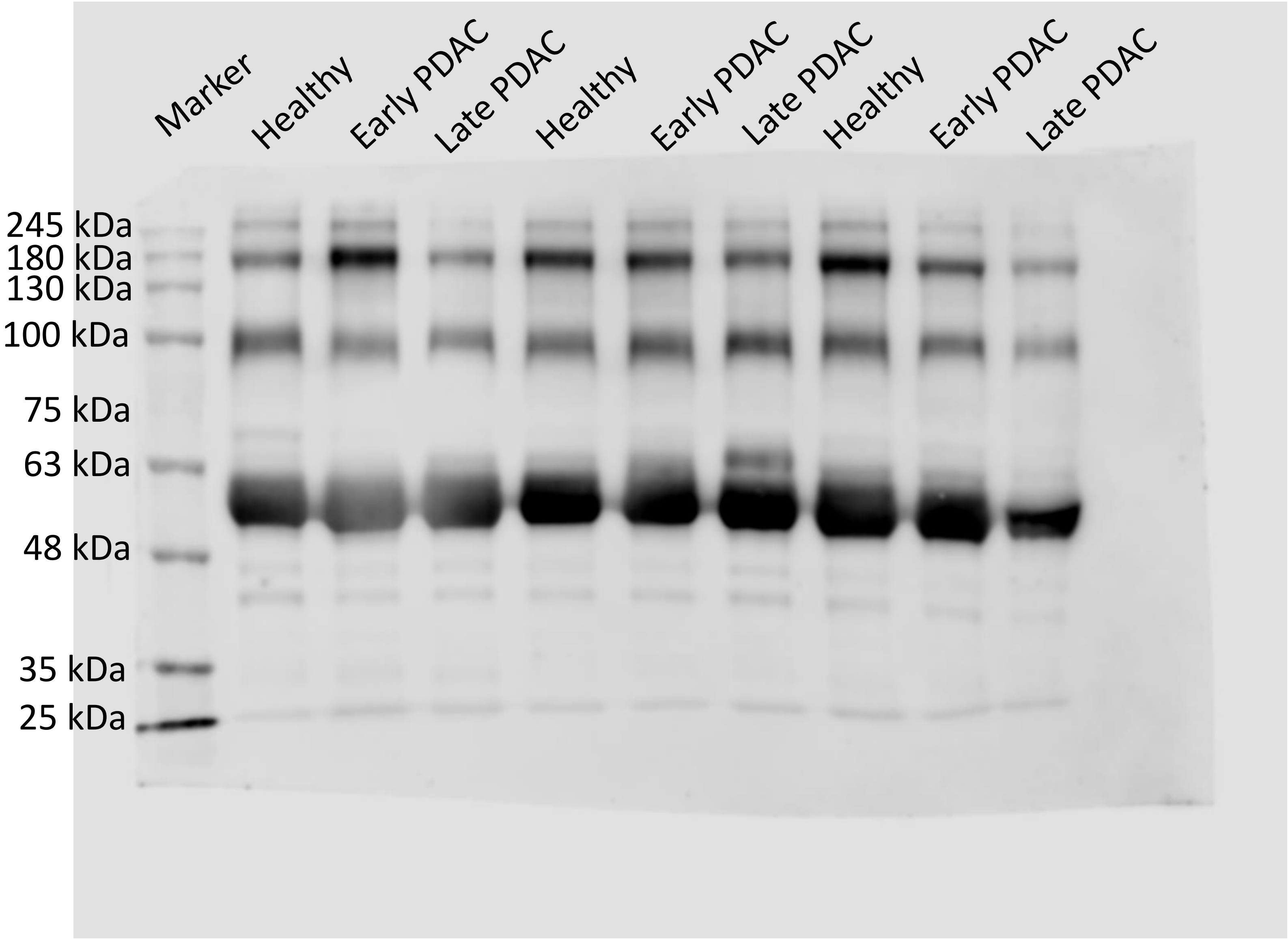

Figure 4A Coomassie

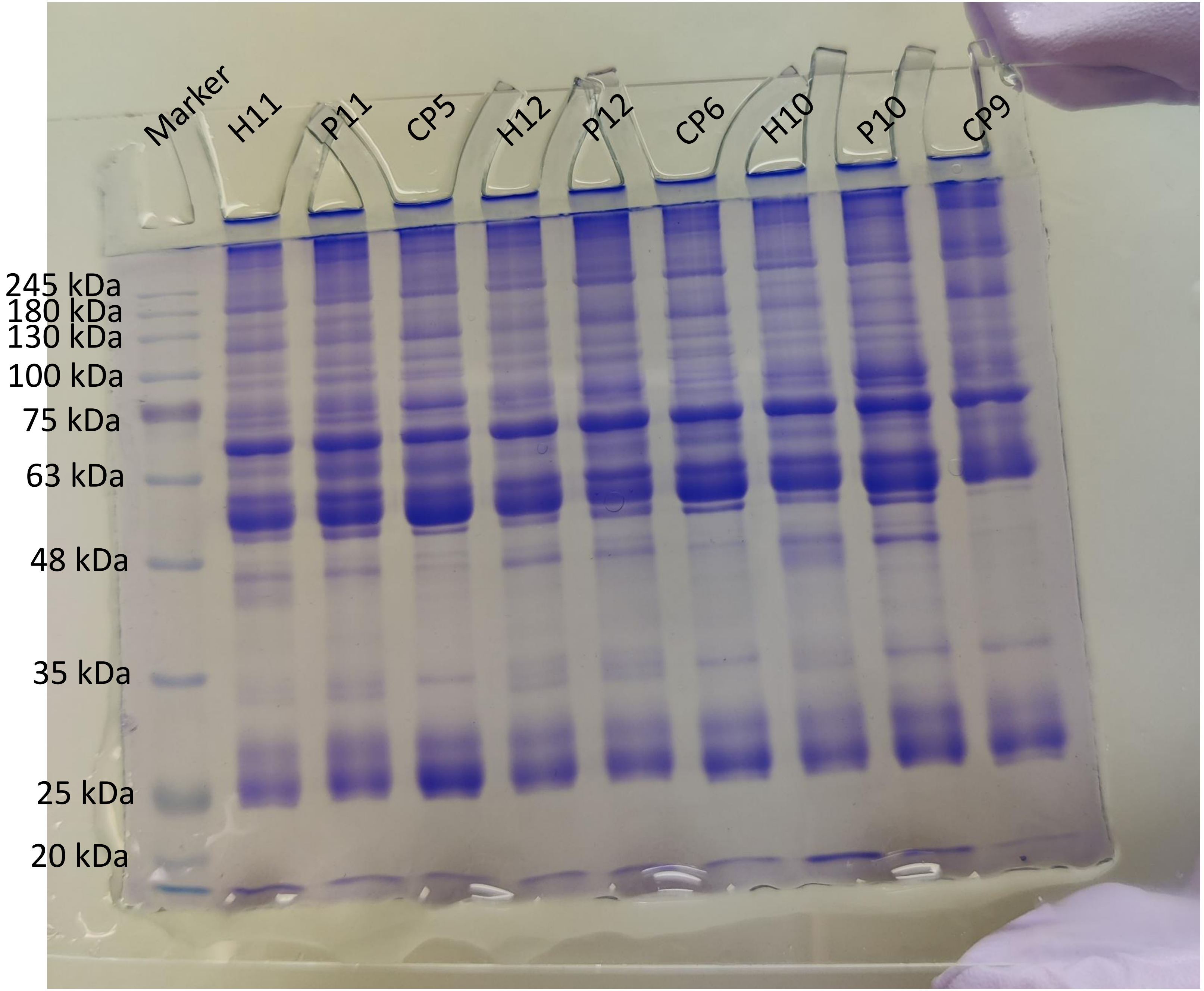

Figure 4A MMA CP

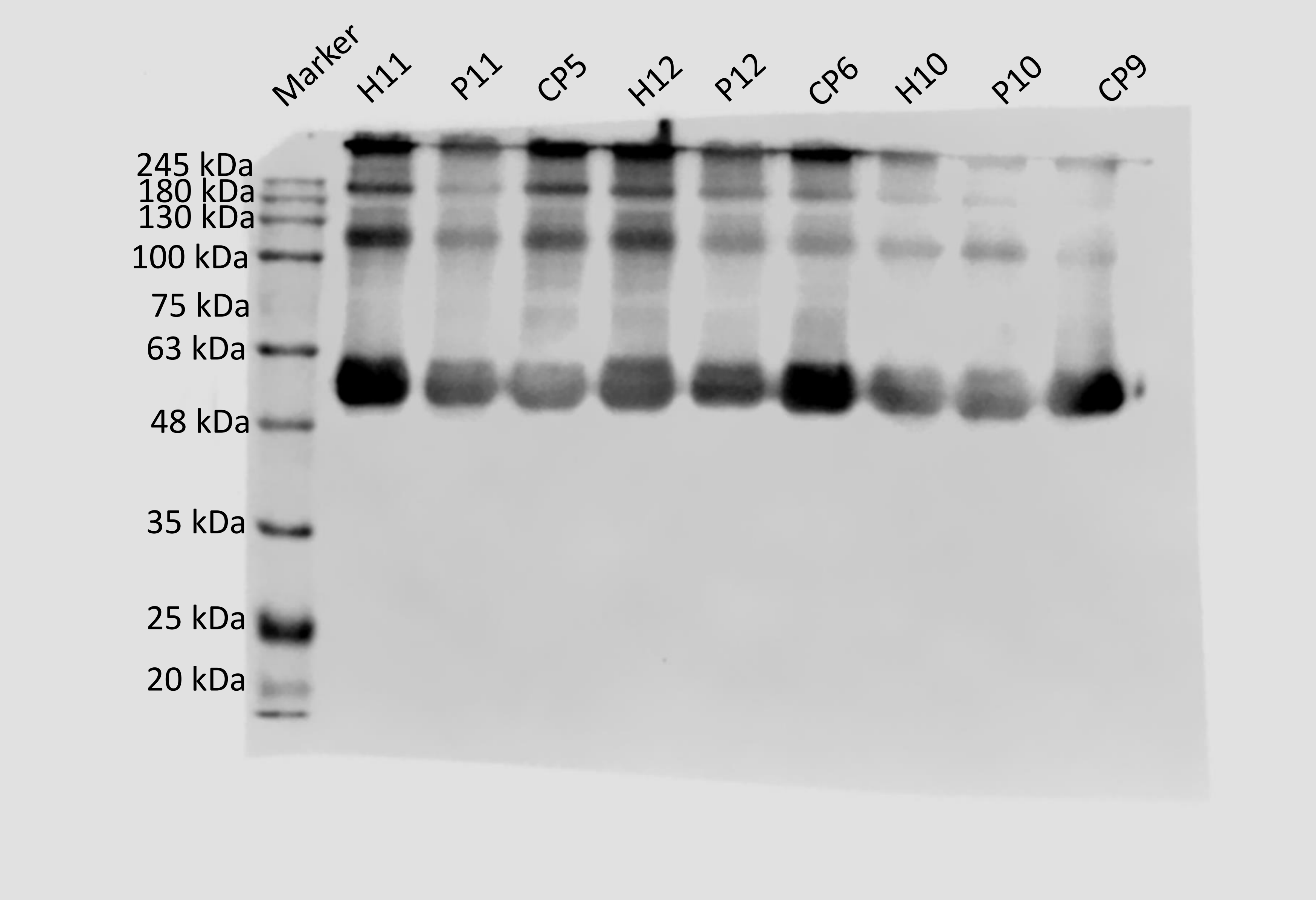

Figure 4A SDMA CP

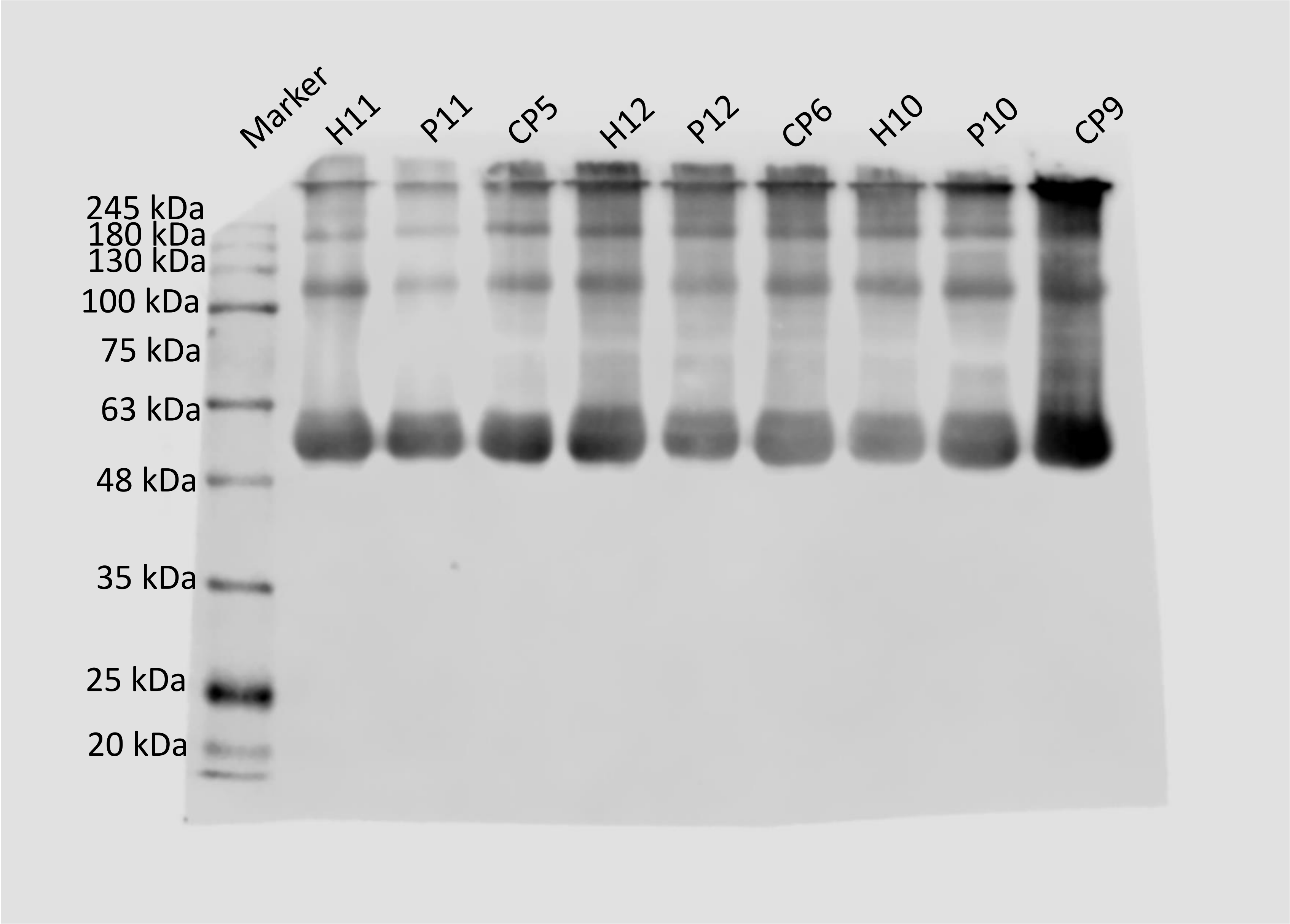

Figure 6C

C3 on SDMA IP

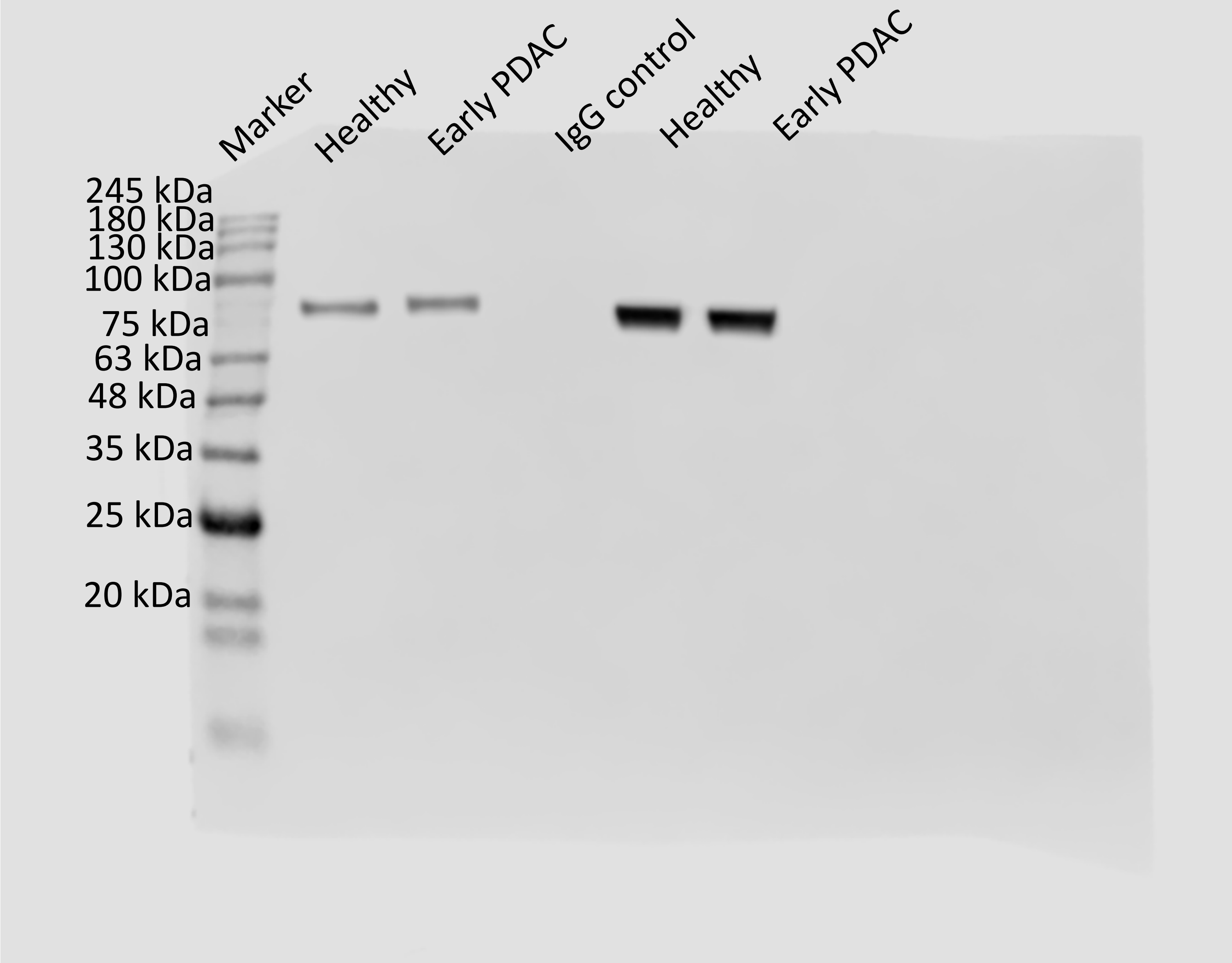

Figure 6C

A2M on SDMA IP

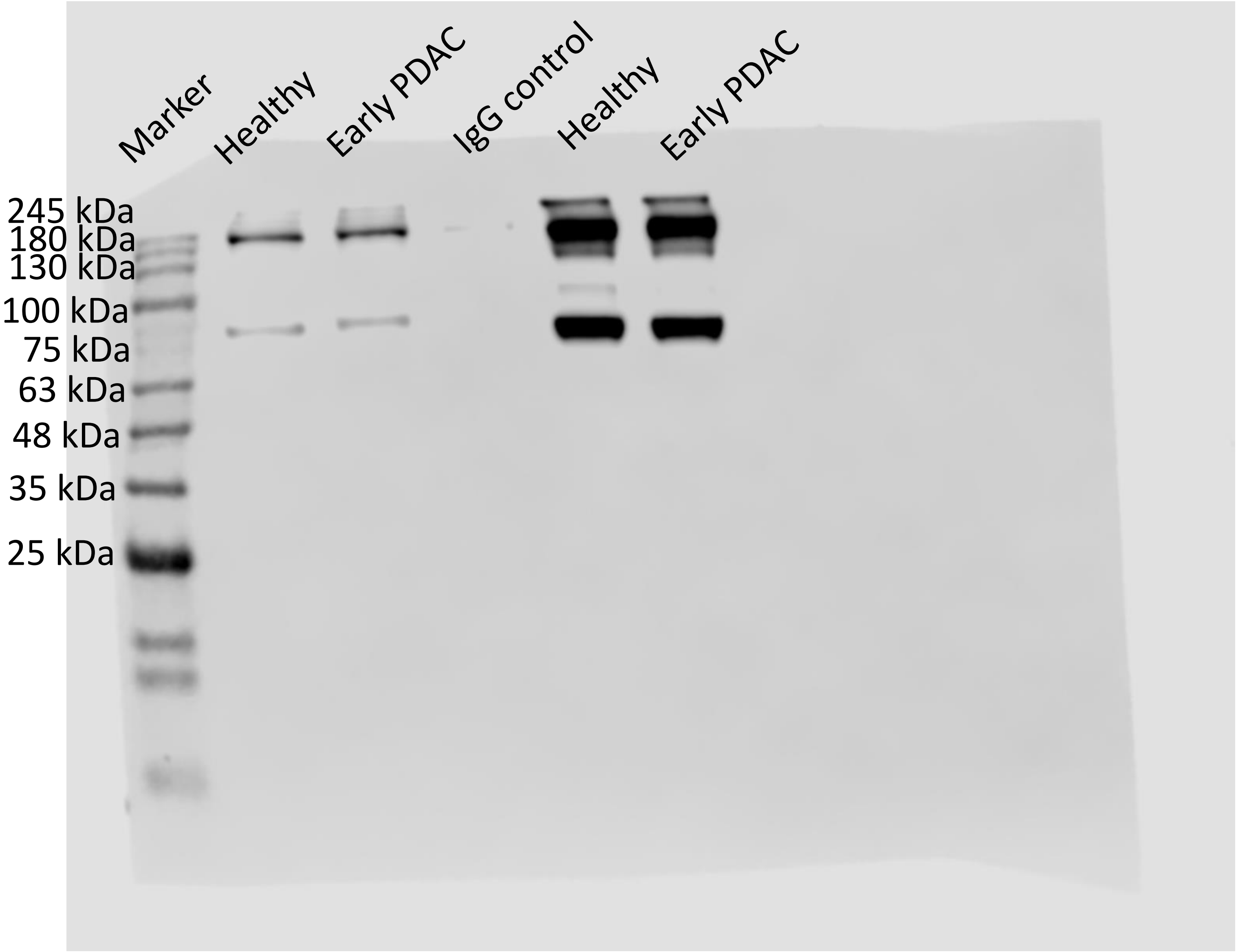

Figure 6D

MMA IP C3

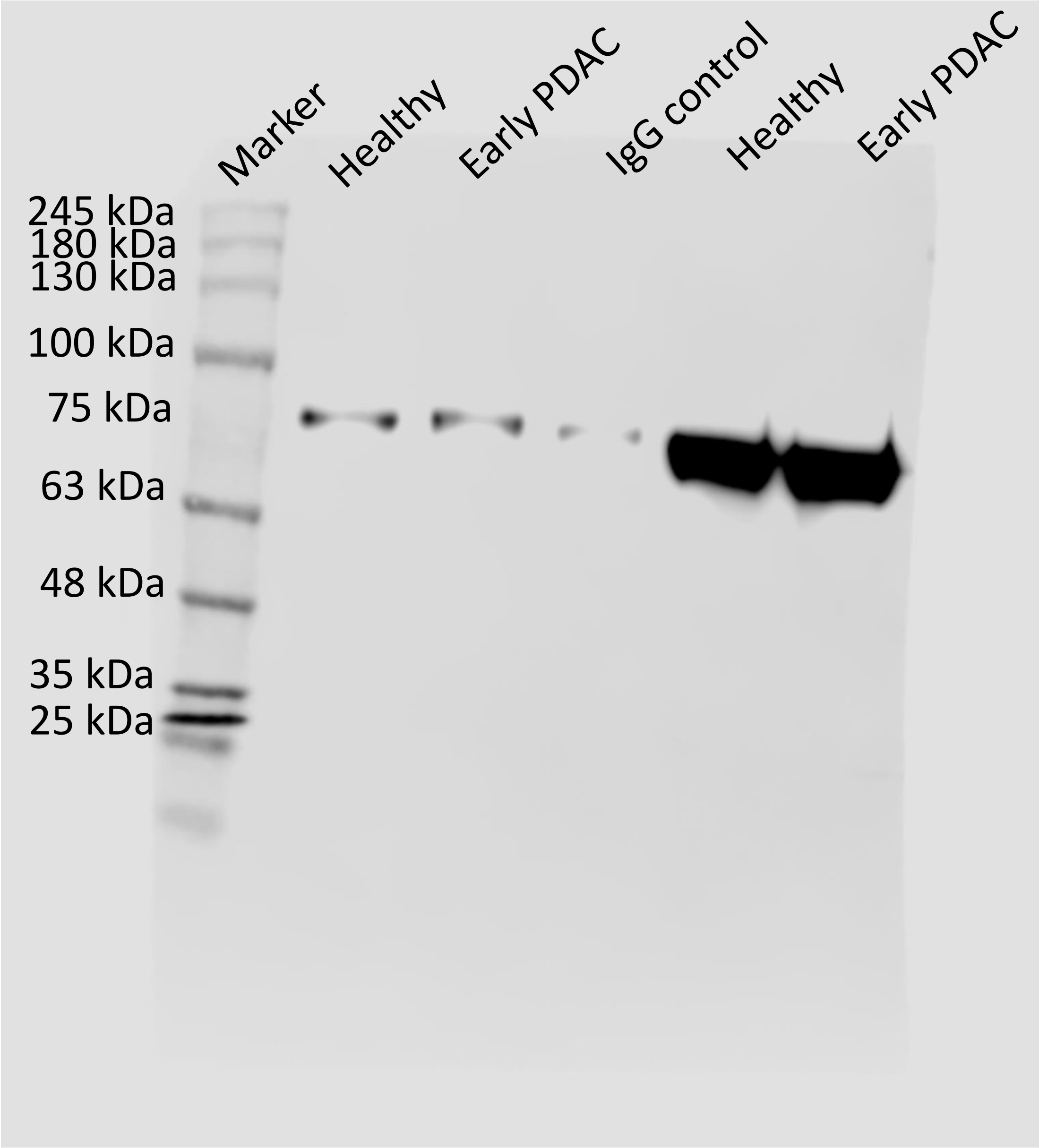

Figure 6D

MMA IP A2M

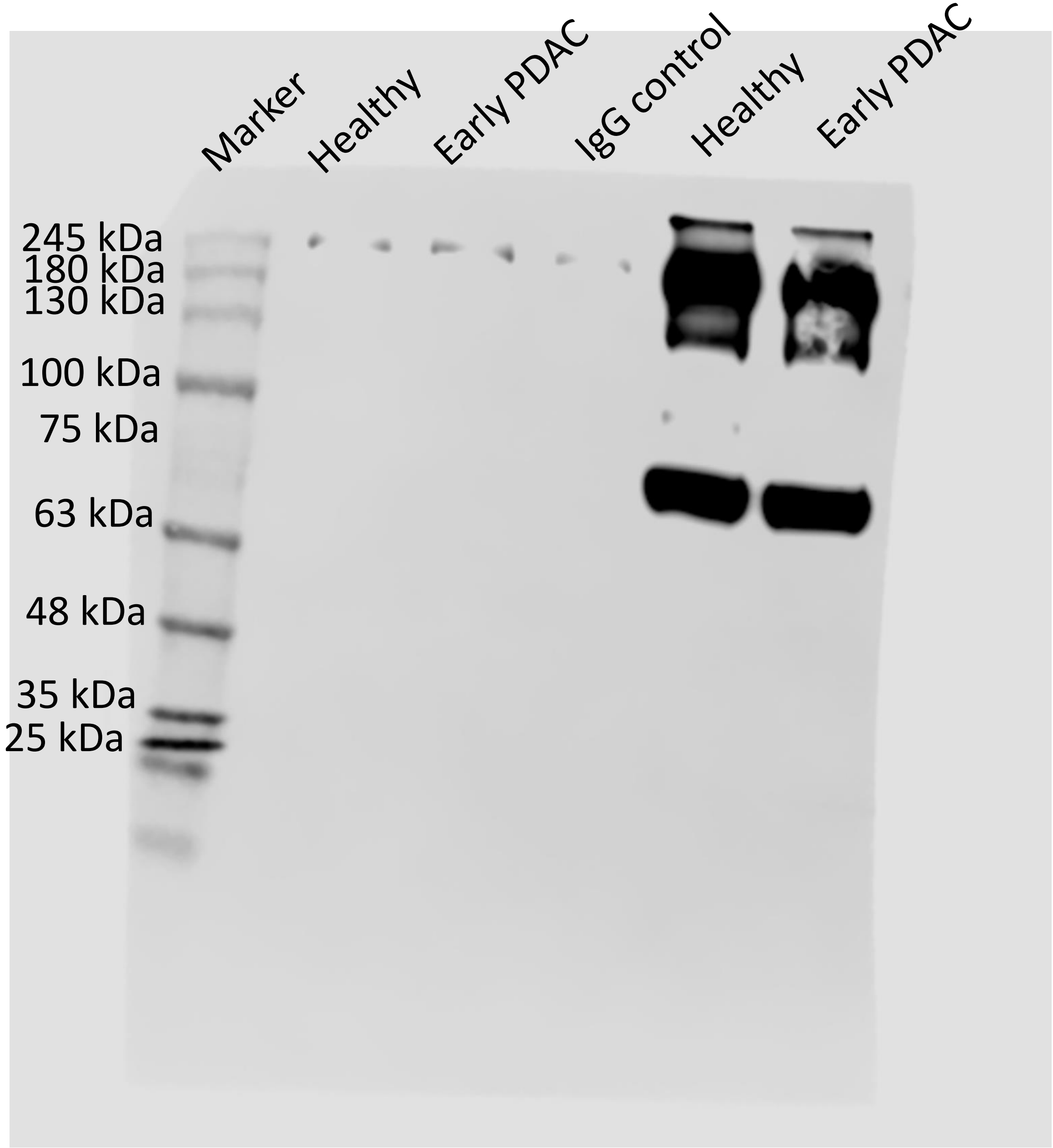

Figure 7A

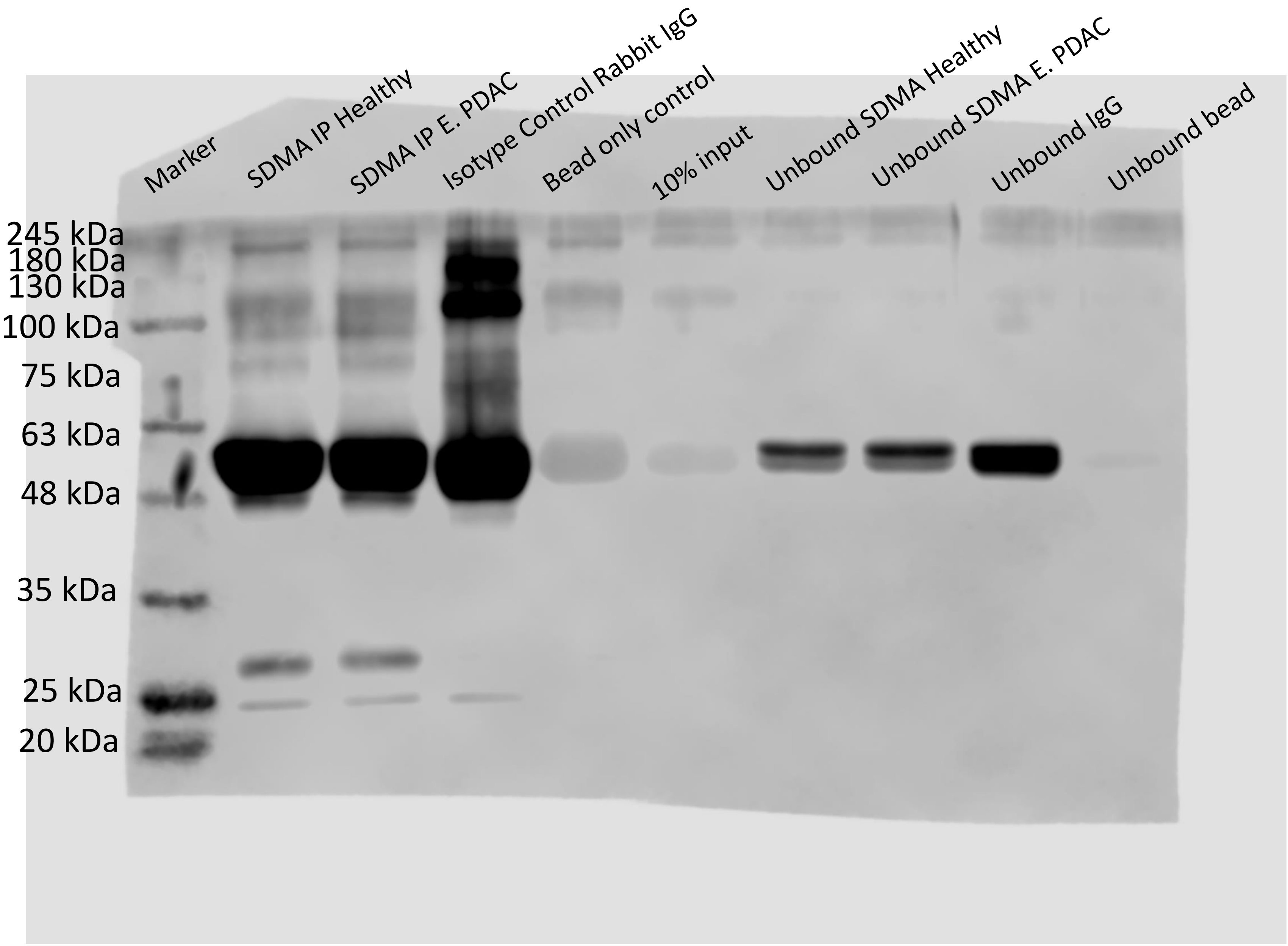
Marker
SDMA IP Healthy
SDMA IP E. PDAC
Isotype Control Rabbit IgG
Bead only control
10% input
Unbound SDMA Healthy
Unbound SDMA E. PDAC
Unbound IgG
Unbound bead
245 kDa
180 kDa
130 kDa
100 kDa
75 kDa
63 kDa
48 kDa
35 kDa
25 kDa
20 kDa
