# Supplementary material for: Protein Arginine Methylation Patterns in Plasma Small Extracellular Vesicles Are Altered in Patients with Early-Stage Pancreatic Ductal Adenocarcinoma
Source: Cancers (Basel). 2024 Feb 3;16(3):654. doi: 10.3390/cancers16030654 (PMC10854811; doi:10.3390/cancers16030654)
Supplement: Supplementary file 1 [file cancers-16-00654-s001.zip › cancers-2827280-supplementary.pdf]

Supplemental File S1.

Supplemental Table S1. Clinicopathological features of the late-stage PDAC patients (*n* = 10, mean age: 66.7, median age: 68).

Plasma Sample	Age	Gender	Race	Tumor stage[a]
LP1	79	Female	CA	4
LP2	66	Female	AA	4
LP3	54	Female	AA	3
LP4	68	Female	AA	3
LP5	68	Female	NA	3
LP6	73	Male	CA	3
LP7	73	Male	CA	3
LP8	73	Male	CA	3
LP9	51	Male	CA	3
LP10	62	Male	CA	3

[a]Pathologic TNM tumor staging, (American Joint Committee on Cancer Care 8th Edition). *CA*: Caucasian; *AA*: African American; *NA*: not available.

Supplemental Table S2. Clinicopathological features of the colon cancer patients (*n* = 16, mean age: 59.75, median age: 58.5).

Plasma Sample	Age	Gender	Race	Tumor stage[a]
C1	72	Female	CA	1
C2	41	Female	CA	3C
C3	53	Female	CA	1
C4	41	Male	CA	3B
C5	55	Male	CA	2A
C6	58	Male	CA	2B
C7	56	Female	CA	1
C8	78	Female	CA	3B
C9	67	Female	CA	3A
C10	58	Female	AA	3B
C11	59	Female	AA	2C
C12	73	Male	CA	NA
C13	69	Male	CA	2
C14	49	Male	CA	NA
C15	67	Male	CA	2A
C16	60	Male	NA	1

[a]Pathologic TNM tumor staging, (American Joint Committee on Cancer Care 8th Edition). *CA*, Caucasian; *AA*, African American; *NA*, not available.

Supplemental Table S3. Clinicopathological features of the chronic pancreatitis patients (*n* = 8, mean age: 53.75, median age: 52).

Plasma Sample	Age	Gender	Race
CP1	53	Male	CA
CP2	51	Male	CA
CP3	51	Male	CA
CP4	46	Female	CA
CP5	56	Female	CA
CP6	66	Female	CA

CP7	47	Male	CA
CP8	60	Male	CA

CA, Caucasian.

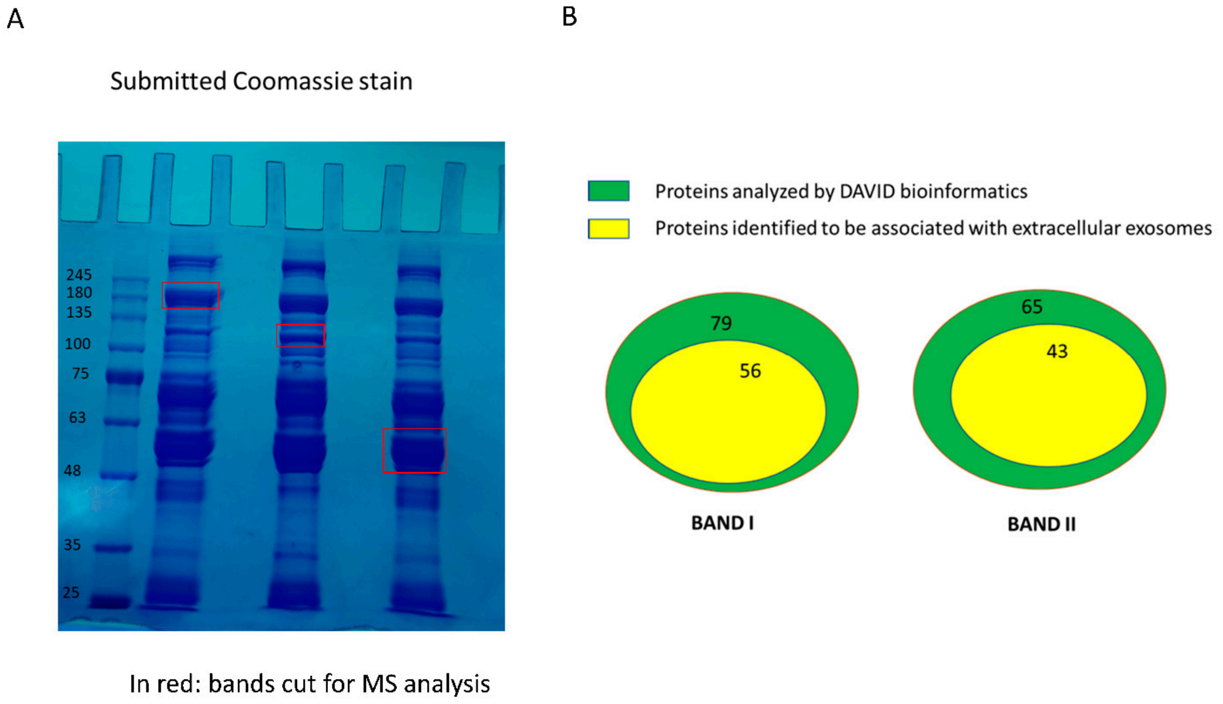

Supplemental Figure S1. Numbers of exosome-associated proteins detected by proteomic analysis of the Coomassie-stained SDMA bands I and II of the plasma sEV lysates. (**A**) Excision of the gel corresponding to SDMA bands I and II for proteomics. (**B**) DAVID bioinformatics analysis of the proteins identified by proteomics.

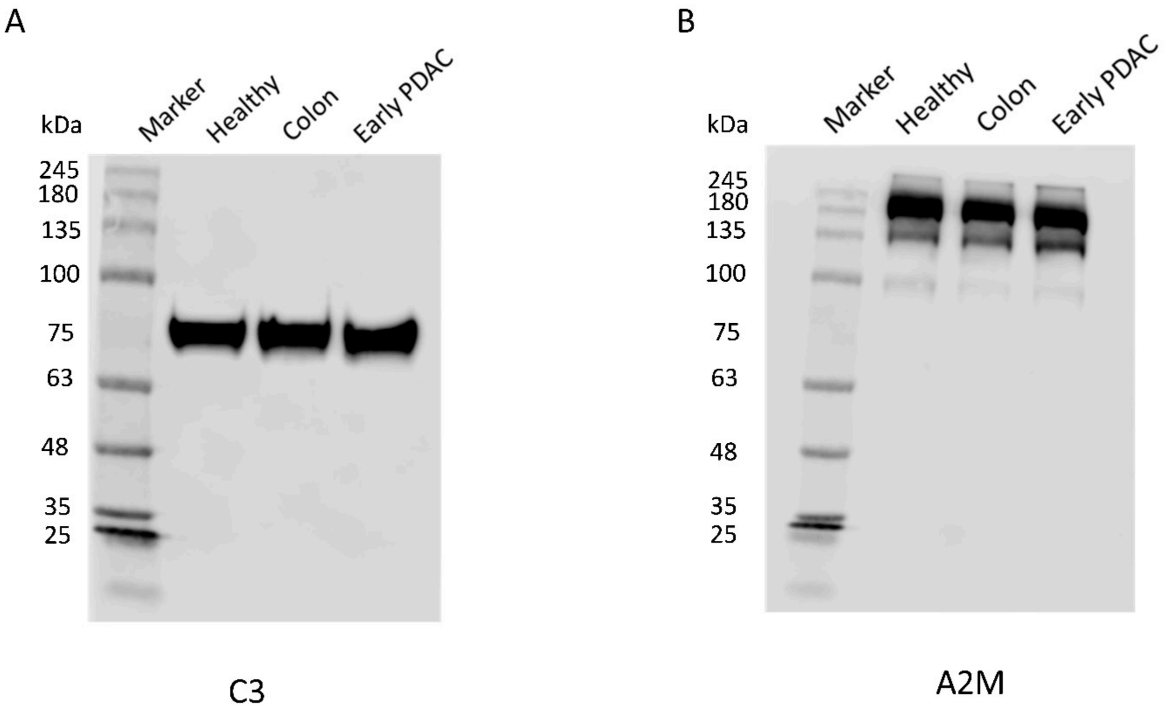

Supplemental Figure S2. Detection of complement C3 and Alpha-2-macroglobulin in plasma sEV lysates. (**A**) Western blot detection of complement C3 in plasma sEV lysates from patients with colon cancer and early-stage PDAC, and healthy controls. (**B**) Western blot detection of Alpha-2-macroglobulin in plasma sEV lysates from patients with colon cancer and early-stage PDAC, and healthy controls.
